# Inflammation and Cancer: Molecular Mechanisms and Therapeutic Targets

**DOI:** 10.1002/mco2.70605

**Published:** 2026-01-31

**Authors:** Xiaodie Liu, Ziyuan Wang, Huirong Zhu, Yicun Han, Qing Ji

**Affiliations:** ^1^ Department of Medical Oncology & Cancer Institute of Integrative Medicine Shuguang Hospital, Shanghai University of Traditional Chinese Medicine Shanghai China; ^2^ Department of Pathology Shuguang Hospital, Shanghai University of Traditional Chinese Medicine Shanghai China

**Keywords:** drug resistance, inflammation, personalized therapy, therapeutic target, tumor

## Abstract

Inflammation is a core pathological factor regulating tumor initiation, progression, and therapeutic resistance, and elucidating its molecular crosstalk with tumors is crucial for developing effective clinical therapies. Internal drivers of inflammation–tumor transformation include genomic disorder, epigenetic memory, mitochondrial stress, and metabolic reprogramming, which synergistically initiate carcinogenesis. External factors amplifying tumor progression cover immune dysfunction, stromal fibrosis, microbial dysbiosis, vascular neoplasia, and neurotoxicity, collectively accelerating tumor development. Notably, current therapies such as immunotherapy and chemoradiotherapy often induce inflammatory accumulation, exacerbating chemoresistance and recurrence. However, cell‐specific inflammatory signal regulation and the precise balance between anti‐inflammatory effects and antitumor efficacy remain understudied, hindering clinical translation of potential strategies. This review systematically organizes the “internal driving force–external attractive force” regulatory network of inflammation‐induced tumors, summarizes preclinical validation of inflammatory targets and combined therapy efficacy, and proposes future focus on cell‐specific inflammatory signal regulation. It fills the gap in systematically integrating inflammation–tumor interaction mechanisms and provides important theoretical/practical guidance for developing precision anti‐inflammatory–antitumor therapies.

## Graphical abstract

Chronic inflammation fuels tumorigenesis via “internal drivers” and “external attractions.” Targeting these core mechanisms with combined therapies/novel deliveries enables precise, potent anti‐inflammatory–antitumor treatment.

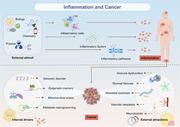

## 1 Introduction

Inflammation is a common pathological response of the body to internal and external stimuli: induced by external factors (e.g., chemicals, physical radiation, biological infections) and internal factors (e.g., metabolism disorders, stress, ischemia, aging) [1]. Essentially, it serves as a protective mechanism of the body [2], typically presenting with “redness, swelling, heat, pain, and dysfunction” [3]. All these responses rely on the secretion of inflammatory factors, activation of inflammatory pathways, and differentiation of inflammatory cells [4], as shown in Table 1. However, long‐term chronic inflammation leads to the persistent accumulation of inflammatory storms, laying the groundwork for tumorigenesis. Rudolf Virchow proposed the hypothesis that “tumors originate from chronic inflammation” in the 19th century [5]. Epidemiological studies have confirmed that approximately 20% of global tumors are associated with chronic inflammation [6], and at the molecular level, clear links have been verified, such as those between chronic hepatitis and liver cancer, ulcerative colitis and colorectal cancer [7, 8, 9]. These findings fully confirm that chronic inflammation is a key risk factor for tumors.

**TABLE 1:** Key inflammatory components in inflammation–tumor transformation: Classification and function.

Category	Subcategory	Core members	Distribution	Core functions	References
Inflammatory cells	Phagocytic cells	Macrophages, neutrophils	Neutrophils (derived from bone marrow hematopoietic stem cells); Macrophages (differentiated from bone marrow‐derived peripheral blood monocytes)	Phagocytosis of pathogens/apoptotic cells; neutrophils release antimicrobial granules	[10, 11]
	Immune‐regulatory cells	T helper 1 (Th1), T helper 2 (Th2), T helper 17 (Th17) cells, dendritic cells (DCs)	Th cells (matured in the thymus); DCs (bone marrow/spleen)	DCs present antigens to activate T cells; Th1 cells combat intracellular bacteria by secreting interferon‐γ (IFN‐γ), Th2 cells resist parasites by secreting interleukin‐4 (IL‐4), and Th17 cells combat fungi by secreting interleukin‐17 (IL‐17)	[12, 13]
	Mediator‐secreting cells	Mast cells, basophils	Mast cells (connective tissue/mucosal layers)	Release histamine/proteases to trigger vasodilation and increased permeability	[14, 15]
Inflammatory factors	Proinflammatory cytokines	Tumor necrosis factor‐α (TNF‐α), interleukin‐6 (IL‐6), and interleukin‐12 (IL‐12)	Macrophages, DCs, neutrophils	Interleukin‐1β (IL‐1β)/TNF‐α induce fever/vascular responses; IL‐6 promotes acute‐phase protein synthesis; IL‐12 drives Th1 differentiation	[16, 17, 18]
	Anti‐inflammatory cytokines	Interleukin‐10 (IL‐10), transforming growth factor‐β (TGF‐β), and interleukin‐35 (IL‐35)	Macrophages, Treg cells, mast cells	IL‐10 inhibits proinflammatory cytokine release; TGF‐β regulates tissue repair and immune tolerance; IL‐35 enhances immune suppression	[19, 20, 21]
	Chemokines	C‐X‐C motif chemokine ligand 8 (CXCL8, also known as interleukin‐8 [IL‐8]), C‐C motif chemokine ligand 2 (CCL2), and C‐X‐C motif chemokine ligand 10 (CXCL10)	Endothelial cells, macrophages, fibroblasts	CXCL8 recruits neutrophils; CCL2 recruits monocytes; CXCL10 recruits Th1 cells	[22, 23, 24]
	Lipid mediators	Prostaglandin E_2_ (PGE_2_) and leukotriene B_4_ (LTB_4_)	Mast cells, macrophages, epithelial cells	PGE_2_ enhances vascular permeability; LTB4 mediates leukocyte chemotaxis	[25, 26]
Inflammatory pathways	Classical proinflammatory pathways	The NF‐κB pathway and mitogen‐activated protein kinase (MAPK, ERK/JNK/p38)	Immune cells and parenchymal cells	NF‐κB regulates transcription of IL‐6/TNF‐α; MAPK transmits pathogen/oxidative stress signals to regulate cell survival	[27, 28]
	Cytokine signaling pathways	Janus kinase‐signal transducer and Activator of transcription (JAK‐STAT) pathway	Immune cells, parenchymal cells (hepatocytes/epithelial cells)	JAK2‐STAT3 mediates IL‐6 signaling; JAK1‐STAT1 mediates IFN‐γ signaling	[29, 30]
	Inflammasome pathways	NOD‐like receptor family pyrin domain‐containing 3 (NLRP3) and absent in melanoma 2 (AIM2) inflammasome pathways	Macrophages, DCs, epithelial cells	Activate caspase‐1 to promote maturation and release of IL‐1β/IL‐18	[31]
	Pathogen recognition pathways	The cyclic GMP–AMP synthase‐stimulator of interferon genes (cGAS‐STING) pathway, TLR pathway	Immune cells, parenchymal cells	cGAS‐STING senses cytosolic DNA to activate type I interferons; TLRs recognize pathogen‐associated molecular patterns (e.g., TLR4 recognizes LPS)	[32, 33]

The association between inflammation and tumors is not a simple pathological superposition but involves a multidimensional dynamic regulatory network, and there remain critical gaps in current research. On one hand, inflammation drives tumorigenesis through the coupling of “endogenous molecular abnormalities” and “exogenous microenvironmental disorders”: via the coupling of “endogenous molecular abnormalities” (e.g., genomic damage by ROS/RNS [34, 35, 36, 37]) and “exogenous microenvironmental disorders” (e.g., immune dysfunction, stromal fibrosis [38]), forming the core “internal drive–external attraction” framework. On the other hand, inflammation exacerbates tumor therapeutic resistance—clinically, chronic inflammation often reduces patients’ sensitivity to immune checkpoint inhibitors, chemotherapy, and radiotherapy [39]. However, the regulatory mechanisms of the “internal drive–external attraction” framework lack systematic organization, and precise intervention strategies for inflammation‐related tumors remain to be improved. With the development of precision medicine, deciphering the core mechanisms of inflammation‐induced tumorigenesis and identifying key regulatory nodes have become urgent needs—this is the core rationale for this review.

This review integrates systematic and innovative approaches around three key dimensions: mechanistic analysis, clinical intervention, and future outlook. Mechanistically, it dissects the “internal drives” (genomic disorder, epigenetic memory, mitochondrial stress, metabolic reprogramming) and “external attractions” (immune dysfunction, stromal fibrosis, microbial dysbiosis, vascular neoplasia, neurotoxicity) of inflammation‐induced tumorigenesis. Clinically, it analyzes inflammation‐mediated drug resistance pathways and summarizes progress in therapeutic target development, auxiliary diagnosis, combination therapy, and novel delivery systems. Its highlight lies in breaking single‐mechanism research limitations and integrating the “inflammation–tumor–resistance” correlative logic.

This review will first systematically dissect the intrinsic molecular mechanisms and extrinsic microenvironmental regulatory factors underlying inflammation‐driven tumorigenesis, then focus on the development of therapeutic targets, clinical research progress, and novel delivery strategies for inflammation‐related tumors, and finally summarize the core conclusions and prospect future research directions. Centered on the core theme of “basic mechanisms–technological innovation–clinical translation,” the full text constructs a comprehensive discussion framework, providing systematic references for the advancement of precision medicine in inflammation‐related tumors.

## 2 Internal Drivers of Inflammation‐to‐Tumor Transformation

The transformation of inflammation to tumor is not an accidentally triggered isolated event, but a progressive process driven by the synergy of multiple intrinsic pathological mechanisms. When the “defensive function” of acute inflammation gets out of control and evolves into chronic inflammation [39], the continuously released inflammatory signals and microenvironmental changes will gradually break down the body's homeostatic defense lines and activate a series of core internal drivers that promote malignant transformation. These internal drivers are intertwined and progressive, ranging from the disruption of genomic stability at the genomic level, the retention of memory at the epigenetic level, to the abnormal remodeling of cellular energy metabolism and organelle functions. Together, they provide the necessary conditions for the transition from inflammation to tumor. In‐depth analysis of the four core internal drivers—genomic disorder, epigenetic memory, mitochondrial stress, and metabolic reprogramming—is key to clarifying the pathological essence of inflammation‐induced tumorigenesis.

### 2.1 Genomic Disorder

As a core link in the body's stress response, the sustained activation of inflammation can drive genomic disorder toward tumor transformation by inducing DNA damage, triggering abnormal activation of oncogenes, and promoting inactivation of tumor suppressor genes. First, chronic inflammation induces DNA damage through multiple mechanisms, laying the initial groundwork for tumorigenesis. Exposure to environmental pollutants is a typical trigger: excessive PM2.5 generates oxidative stress by activating pathways such as the ROS‐DNMT pathway, while long‐term accumulation of asbestos fibers causes persistent inflammation‐mediated DNA damage. These two factors increase the risk of lung cancer [40] and pleural mesothelioma [41], respectively. Endogenous metabolic abnormalities also play a role. Oxidative stress induced by hyperuricemia directly causes DNA damage and genomic instability, creating a proinflammatory microenvironment that accelerates inflammation–cancer transformation [42]. In addition, the abnormal formation of R‐loops (noncanonical nucleic acid structures) can activate innate immune pathways through DNA damage induction, triggering inflammatory signal cascades in the tumor microenvironment and further amplifying genomic damage effects [43]. Pathogens such as *Escherichia coli* can also directly induce DNA damage in mammalian cells [44], serving as potential initiating factors for digestive tract tumors such as colorectal cancer.

Abnormal activation of inflammatory signaling pathways is a key driver of oncogene dysfunction. As a core transcription factor regulating inflammation, dysregulated activation of NF‐κB can transactivate proinflammatory factor‐encoding genes and promote oncogene expression through crosstalk with other pathways, thereby driving tumor cell proliferation, metastasis, and therapeutic resistance [45]. In colorectal cancer, NF‐κB activation promotes the binding of P65 to the TRIM31 promoter, upregulating its transcription. TRIM31 then stabilizes the YBX1 protein through ubiquitination modification, forming an “NF‐κB‐TRIM31‐YBX1” positive feedback loop that accelerates inflammation‐induced carcinogenic transformation [46]. In lung cancer progression, RBM14 forms a complex with p23 to enhance its transcriptional activity, regulating CXCL1 expression to promote the epithelial–mesenchymal transition (EMT) process. This process is closely associated with cytokine‐mediated signal activation in the inflammatory microenvironment [47]. In an alcoholic pancreatitis model, persistently activated CREB directly drives pancreatic tumor progression by reprogramming acinar cells and enhancing profibrotic inflammation, highlighting the critical role of inflammation‐driven oncogene activation in tumorigenesis [48].

Beyond the above, chronic inflammation can inhibit the function of tumor suppressor genes through multiple means, impairing the ability to maintain genomic homeostasis. The genomic landscape of pleural mesothelioma is characterized by alterations in tumor suppressors; inactivation of tumor suppressor genes such as BAP1, CDKN2A, and TP53 is closely associated with asbestos‐induced chronic inflammation [41]. In pancreatic cancer, risk‐related low KLHL17 expression is associated with activation of proinflammatory pathways. As a member of the Cullin‐E3 ubiquitin ligase complex, KLHL17 can alleviate cell damage and inflammation by degrading nestin and vimentin; loss of its function is equivalent to the loss of a tumor‐suppressive barrier [49]. In acute myeloid leukemia, the p30 isoform produced by mutant CEBPA downregulates the function of Activator Protein‐1 (AP‐1) family proteins, reducing the expression of inflammatory genes and rendering cells tolerant to inflammatory stress. This indirectly impairs the immune surveillance function associated with tumor suppression [50]. Additionally, NOTCH1 reduces Nrf2 stability through interaction with KEAP1. As a key factor in antioxidant defense and cell protection, inhibited Nrf2 function exacerbates liver damage and inflammation, indirectly promoting hepatocellular carcinoma development—equivalent to weakening a potential tumor‐suppressive defense mechanism [51]. By inducing DNA damage, driving abnormal activation of oncogenes, and promoting inactivation of tumor suppressor genes—three progressive key links—inflammation systematically breaks down the homeostatic defense lines of the genome, constructing a pathological chain of “damage initiation–abnormal driving–defense deficiency.” This chain not only lays the core material foundation for the transformation of inflammation to tumor but also serves as the most fundamental molecular cornerstone among the internal drivers of inflammation‐induced tumorigenesis.

### 2.2 Epigenetic Memory

Sustained activation of inflammation can disrupt genomic functional homeostasis through multiple epigenetic regulatory mechanisms, laying a core foundation for tumorigenesis. This primarily involves four key dimensions: DNA methylation, histone modification, noncoding RNA regulation, and RNA methylation with chromosomal remodeling [52]. The association between inflammation and abnormal DNA methylation is universal across cancer types; prior inflammation accumulation drives malignant transformation through DNA methylation remodeling. In colorectal cancer, DSS‐induced intestinal inflammation promotes the accumulation of modifying enzymes such as DNA methyltransferase 1 (DNMT1) and protein arginine methyltransferase 6 (PRMT6), which synergistically inhibit the expression of tumor suppressor genes (e.g., Cdkn1a, Cdkn1b) through DNA methylation and histone modification [53]; in ulcerative colitis‐associated colorectal cancer (CAC), hypermethylation of the promoters of PIGR and HNF4a genes disrupts the epithelial barrier, exacerbates inflammation, and impairs the function of EMT negative regulators [54]. In a liver cancer model, inflammatory factors such as NO and PGE_2_ disrupt DNA hypomethylation by regulating DNMT1 stability, activating LINE‐1 retrotransposons and causing genomic instability [55]. During gastric cancer development, inflammation‐highly expressed PGE_2_ enhances DNA methylation via the cyclooxygenase‐2 (COX‐2) pathway, inhibiting tumor suppressor genes such as MGMT and CNR1 and accelerating the progression of intestinal metaplasia [56].

The plasticity of barrier epithelial cells is crucial in inflammation‐induced tumorigenesis. “Memory” of histone modifications retained after inflammation resolution can increase tumor risk over the long term. In a pancreatitis model, acinar cells maintain high histone H3 lysine 4 monomethylation (H3K4me1) expression at metaplastic genes even after inflammation resolution. Although this epigenetic memory enables response to secondary damage, it significantly increases the probability of *Kras* gene mutations [57]; when pancreatic epithelial cells are exposed to inflammatory and oncogenic stress, decreased MEN1 expression inhibits H3K4me. Combined with enhanced DNA methylation, this downregulates tumor suppressor genes such as CDKN1B and CDKN2C to promote unlimited proliferation [58]. In colorectal cancer, C1Q^+^TPP1^+^ tumor‐associated macrophages (TAMs) upregulate CEBPD by secreting factors such as IL‐6, prompting it to bind to the promoter of the histone methyltransferase SETD8 to enhance transcription. SETD8‐mediated monomethylation of p53K382 inactivates p53 function. This modification is only present in colorectal cancer stem cells and specific macrophages and is closely associated with poor patient prognosis [59]. As a key bridge between inflammation and histone modification, NF‐κB can bidirectionally regulate gene expression: in non–small‐cell lung cancer, it inhibits H3K9 acetylation at the GPRC5A promoter while activating the oncogenic MUC1‐C protein by upregulating H3K4me3 modification, driving malignant transformation [60].

Noncoding RNAs in the inflammatory microenvironment interfere with gene function by targeting nucleic acids or proteins, serving as important mediators of inflammation–cancer transformation. In liver inflammation, increased miR‐873‐5p expression promotes hepatocyte apoptosis [61], while elevated miR‐378 exacerbates inflammatory fibrosis; the long noncoding RNA lnc‐Helf binds to PTBP1 to activate the AKT pathway, enhancing the activation of hepatic stellate cells and aggravating inflammatory damage [62]. In the intestine, deletion of the long noncoding RNA EPR triggers excessive epithelial proliferation and inflammatory infiltration, significantly increasing susceptibility to DSS‐induced tumors. The mechanism is associated with oncogenic transcriptome rearrangement [63]. In esophageal squamous cell carcinoma, circTMEM45A is upregulated in tumor tissues. It stabilizes IL1B mRNA by binding to U2AF2 and acts as a protein scaffold to enhance the interaction between ELAVL1 and IL1R1 mRNA, activating the IL‐1β/IL1R1 proinflammatory cascade and driving tumor progression [64].

Abnormal RNA methylation and chromosomal structure remodeling form a synergistic regulatory network in inflammation–cancer crosstalk. In esophageal squamous cell carcinoma, circTMEM45A interacts with the m5C methyltransferase NSUN2 and readers ALYREF and YBX1, promoting the nuclear export and stability of NLRP3 mRNA and activating the NLRP3/caspase‐1/IL‐1β inflammatory pathway. This highlights the protumor role of crosstalk between RNA methylation and noncoding RNAs [64]. In oral cancer, imbalanced regulation between the NuRD and SWI/SNF chromatin remodeling complexes controls inflammation and EMT: deletion of CDK2AP1 triggers a proinflammatory secretome containing multiple chemokines and cytokines, recruiting monocytes and differentiating them into M2‐like macrophages. This drives malignant progression by remodeling the tumor microenvironment [65]. The NF‐κB‐activated MUC1‐C oncogene is a core regulatory node: on the one hand, it recruits the PBAF chromatin remodeling complex to increase chromatin accessibility in regulatory regions of oncogenes such as TDP43 and NEAT1, driving their transcription and maintaining cancer stem cell status and drug resistance [66]; on the other hand, it inhibits the YTHDF2‐mediated RNA degradation pathway, enhancing the stability of procancer RNAs such as XIST and ultimately inactivating tumor suppressor genes [67]. The four epigenetic mechanisms—abnormal DNA methylation, “inflammatory memory” of histone modifications, noncoding RNA regulation, and RNA methylation with chromosomal remodeling—are intertwined to form a network. Inflammation disrupts genomic homeostasis by perturbing this network, laying a critical foundation for tumorigenesis. Deciphering its logic can provide precise directions for targeted therapy.

### 2.3 Mitochondrial Stress

Chronic inflammation initiates mitochondrial stress through dual pathways of direct damage and signal regulation, laying a critical hidden danger for tumorigenesis. Oxidative stress, a core pathological state of inflammation, leads to excessive accumulation of reactive oxygen species that directly damage mitochondrial DNA (mtDNA), disrupt functional homeostasis, and trigger cellular senescence. Senescent cells secrete the senescence‐associated secretory phenotype (SASP), which further amplifies local inflammation, forming an initial pathological cycle of “inflammation–mitochondrial damage–senescence–inflammation” [68]. This mechanism is particularly typical in the transformation of chronic obstructive pulmonary disease (COPD) to non–small‐cell lung cancer: disrupted mitophagy pathways in COPD patients lead to the accumulation of dysfunctional mitochondria, which not only exacerbate oxidative stress but also activate inflammasomes, significantly increasing carcinogenic risk through persistent inflammation [69]. In addition, fecal microbiota transplantation experiments in aged mice confirmed that proinflammatory microbiota can upregulate colon mitochondrial‐related genes and abundance, accompanied by increased levels of inflammatory factors such as IL‐6 and TNF‐α. This ultimately triples the incidence of colon tumors [70].

In the tumor microenvironment, abnormally secreted cytokines can interfere with mitochondrial function through signaling pathways, creating conditions for tumor immune escape. The infection process of hepatitis C virus (HCV) embodies a similar logic: HCV proteins specifically bind to mitochondria‐associated endoplasmic reticulum membranes (MAMs). Although this does not affect calcium signaling or glucose homeostasis, it supports viral replication by disrupting mitochondrial structural integrity. Over the long term, the combination of inflammation and mitochondrial stress can promote hepatocellular carcinoma development [71]. In alcohol‐related liver disease, abnormal expression of MCJ—a negative regulator of mitochondrial respiration—exerts organ‐dependent effects: systemic MCJ deficiency exacerbates increased intestinal permeability and endotoxemia, aggravating liver damage through systemic inflammation and indirectly driving the transformation of liver disease to tumor [72]. Mitochondrial stress is not isolated damage; the released damage signals further exacerbate inflammation, forming a vicious cycle that drives tumor progression. mtDNA release is a key node connecting mitochondrial stress and inflammation: loss of VDAC2 in tumor cells relieves inhibition of mtDNA release. Free mtDNA activates the cGAS‐STING pathway, which enhances tumor sensitivity to interferon γ while clearly revealing the core mechanism of mitochondrial damage‐mediated inflammatory signal activation [73]. Studies on doxorubicin‐induced cardiotoxicity have also confirmed this pathway: the drug causes opening of the mitochondrial permeability transition pore and mtDNA leakage, activating the cGAS‐STING pathway and driving inflammation and myocardial senescence. Myocardial cell‐specific knockout of STING can partially rescue cardiac dysfunction [74]. In the progression of pulmonary fibrosis to tumor, oxidative damage inhibits the SENP1–Sirt3 axis in AT2 cells, leading to massive accumulation of mitochondrial reactive oxygen species (mtROS). This not only triggers cell apoptosis but also exacerbates lung tissue inflammation and fibrosis, providing a proinflammatory microenvironment for subsequent tumorigenesis [75].

Targeted intervention of the mitochondria–inflammation–tumor axis has become a potential therapeutic strategy to break the pathological cycle. In radiotherapy combination therapy, regulation of mitochondrial energy metabolism exhibits dual advantages: IR‐TAM@Alb nanoparticles reverse tumor hypoxia and downregulate programmed death‐ligand 1 (PD‐L1) and TGF‐β by targeting inhibition of oxidative phosphorylation, sensitizing tumors to radiotherapy. Simultaneously, they improve radiotherapy‐induced pulmonary fibrosis by inhibiting TGF‐β, avoiding inflammation‐related treatment side effects [76]. In the prevention and treatment of doxorubicin cardiotoxicity, mitochondrial supplementation can reduce mtDNA leakage and cGAS‐STING pathway activation, providing ideas for improving the safety of cancer chemotherapy and indirectly ensuring the effectiveness of antitumor therapy. These studies indicate that precise regulation of mitochondrial function can simultaneously interfere with inflammatory and tumor processes, holding broad clinical prospects [73].

### 2.4 Metabolic Reprogramming

Chronic inflammation can directly trigger abnormal reprogramming of cellular metabolic programs by activating key signaling pathways or remodeling the microenvironment, providing initial momentum for tumorigenesis. In early‐onset colorectal cancer, hyperinsulinemia associated with insulin resistance overlaps with chronic inflammation. Elevated insulin‐like growth factor levels further activate inflammatory pathways, driving tumor development by regulating oncogene expression and antiapoptotic programs [77]. In pancreatic cancer, chronic inflammation‐induced IL‐22 signaling enhances *Kras* mutation‐mediated STAT5 phosphorylation. Activated STAT5 not only directly binds to the promoters of metaplastic mediators such as HNF1β to promote precancerous lesions but also supports the progression of inflammation‐driven pancreatic ductal adenocarcinoma (PDAC) by maintaining the energy metabolic homeostasis of tumor cells [78]. In *KRAS*‐mutant lung adenocarcinoma, SPHK1 deletion disrupts lipid homeostasis. Accumulation of abnormal lipid metabolites significantly exacerbates local inflammation, recruiting more TAMs and forming a positive feedback loop of “metabolic disorder‐enhanced inflammation–tumor progression” that accelerates disease progression [79].

Metabolic reprogramming is not merely an adjustment of energy supply; it can further enhance inflammatory responses through the accumulation of metabolites or changes in enzyme activity, accelerating tumor progression. In colorectal inflammatory carcinogenesis, DSS‐induced inflammation activates IP6K2 via the ROS–Src phosphorylation axis, prompting it to synthesize the tumor metabolite 5‐IP7. This metabolite inhibits inositol 5‐phosphatase activity, promotes PI(4,5)P2‐mediated endocytic adaptor recruitment, and ultimately triggers E‐cadherin endocytosis and β‐catenin activation, disrupting intestinal epithelial barrier homeostasis [80]. In pleural mesothelioma, dysregulated NF‐κB pathway drives high FABP5 expression. As a key molecule in lipid metabolism, FABP5 mediates the intracellular accumulation of fatty acids such as myristic acid while amplifying NF‐κB‐dependent inflammatory responses, forming a vicious cycle of “inflammation–abnormal lipid metabolism” [81]. During breast cancer brain metastasis, IKKβ/IKKα imbalance in the NF‐κB pathway initiates dual metabolic reprogramming: on the one hand, it disrupts the blood–brain barrier by secreting chemokines such as IL‐8; on the other hand, it upregulates glutamate transporters EAAT1/2, enabling tumor cells to utilize abundant glutamate in the brain as an energy source to support their invasive colonization [82].

The close association between metabolic reprogramming and inflammation provides new targets for tumor therapy; targeted regulation of their interaction axis can effectively inhibit tumor development. Huangqin Decoction remodels arachidonic acid metabolism by regulating the intestinal microbiome, targeting the key metabolic enzyme ALOX12 to inhibit colorectal inflammatory carcinogenesis. Its protective effect can be transmitted through intestinal microbiota transplantation, confirming the feasibility of metabolic intervention [83]. In colorectal cancer, epithelial DHPS reduces oxidative damage by supporting the translation of electrophile detoxification enzymes; supplementation with spermidine enhances DHPS activity to exert chemopreventive effects. Meanwhile, IP6K2 isoform‐selective inhibitors protect the intestinal epithelial barrier against inflammation and cancer by reducing 5‐IP7 synthesis [84]. Both show clear antitumor effects, providing practical evidence for precise intervention of the metabolism–inflammation axis. Chronic inflammation triggers cellular metabolic reprogramming through signaling pathway activation and microenvironment remodeling, providing initial momentum for tumorigenesis. Moreover, reprogramming conversely exacerbates inflammation and tumor progression, forming a protumor vicious cycle.

**FIGURE 1:**
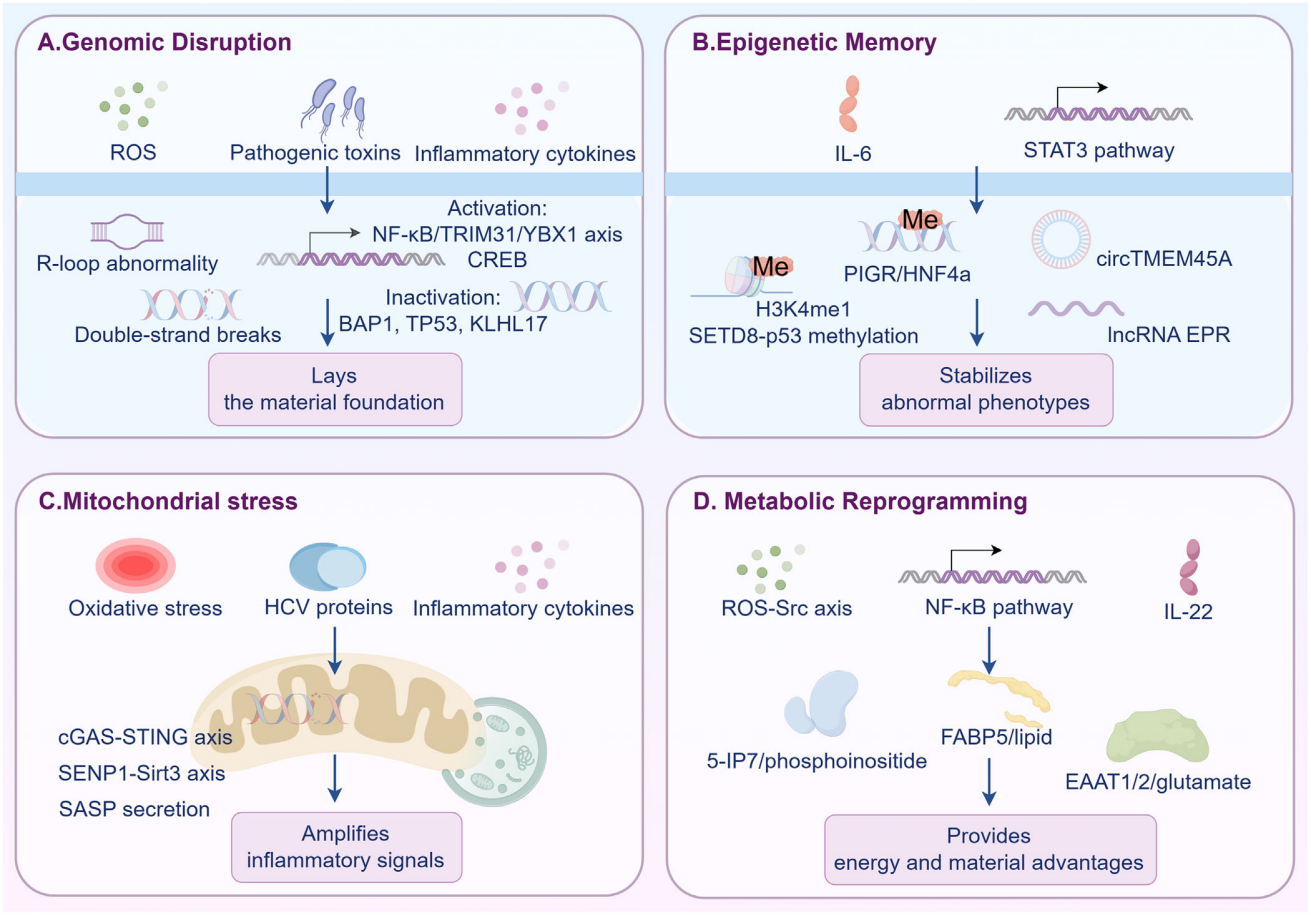
Intrinsic drivers of inflammation‐to‐cancer transition. Core mechanism: Genomic disorder, epigenetic memory, mitochondrial stress, and metabolic reprogramming form a synergistic “damage–memory–stress–remodeling” network to promote inflammation‐to‐tumor transformation.

Genomic disorder, epigenetic memory, mitochondrial stress, and metabolic reprogramming constitute the four core internal drivers of inflammation‐to‐tumor transformation, as shown in Figure 1. They do not act independently but form a synergistic network of “damage–memory–stress–remodeling”: genomic disorder lays the material foundation for malignant transformation; epigenetic memory solidifies inflammation‐related abnormal phenotypes; mitochondrial stress amplifies pathological effects through signal transmission; and metabolic reprogramming provides survival advantages for tumor cells. The dynamic interaction of these four internal drivers completely shifts inflammation from “physiological defense” to “pathological tumorigenesis.” Clarifying the mechanisms and associated logic of these internal drivers not only deepens understanding of the pathological process of inflammation‐induced tumorigenesis but also provides precise molecular targets and strategic directions for targeted intervention of inflammation‐related tumors.

## 3 External Attractions of Inflammation‐to‐Tumor Transformation

The malignant progression of inflammation to tumor relies not only on the drive of intrinsic cellular pathological mechanisms but also on the synergistic “pull” of multiple external factors in the tumor microenvironment. Under chronic inflammation, the local microenvironment undergoes gradual pathological remodeling: the physiological structure and functional network that originally maintain tissue homeostasis are disrupted, giving rise to a series of “external attractions” that promote tumor occurrence and development. These include immune dysfunction (which collapses the antitumor surveillance barrier), stromal fibrosis (which builds a physical scaffold for tumor invasion), microbial dysbiosis (which disturbs inflammatory balance), vascular neoplasia (which provides nutritional support for tumors), and neurotoxicity (which exacerbates microenvironmental disorder through signal regulation). These external attractions are interrelated and function collectively, creating a suitable “soil” for the transformation of inflammation to tumor, and represent a crucial dimension for deciphering the comprehensive mechanism of inflammation‐induced tumorigenesis.

### 3.1 Immune Dysfunction

Chronic inflammation initiates immune dysfunction through signal regulation, affecting cell differentiation and functional expression. Inflammation caused by peripheral ischemia activates the NLRP3 inflammasome in the bone marrow, driving hematopoietic stem cells toward CD150^+^ myeloid‐biased differentiation. This leads to increased output of monocytes and neutrophils while reducing lymphocyte production. Long‐term reprogramming of this hematopoietic pattern directly skews immune balance, creating initial conditions for a proinflammatory and tumor‐permissive microenvironment [85]. Systemic inflammation induced by tumors also undergoes epigenomic priming of circulating monocytes, extensively remodeling the H3K4me3 promoter and H3K4me1 enhancer landscapes. This process inhibits interferon response regulatory elements while constructing enhancers containing binding motifs for proinflammatory transcription factors such as C/EBP and AP‐1, “imprinting” these monocytes with a protumor gene expression program before they infiltrate the tumor [86]. Additionally, signal crosstalk between TNF and type I interferons regulates the fate transition of plasmacytoid dendritic cells (pDCs): TNF promotes pDCs to lose IFN‐I secretion capacity and differentiate into conventional dendritic cells (cDCs), while type I interferons block this process. This imbalanced transition is prevalent in inflammatory diseases and aging, directly impairing innate immune defense functions [87].

Inflammation‐induced immune cell dysfunction and cytokine network imbalance form a positive feedback loop to reinforce immune dysfunction. TAMs are core effector cells: SPP1^+^ macrophages secrete TNF‐α and IL‐1β via the NF‐κB pathway, bidirectionally upregulating OPN expression in both themselves and tumor cells. This forms a cycle of “cytokine secretion–OPN upregulation–inflammation enhancement,” directly promoting the proliferation of head and neck squamous cell carcinoma [88]; Activation of the SENP1–Sirt3 axis drives mitochondrial metabolic reprogramming in TAMs, enhancing cholesterol and acetyl‐CoA synthesis, promoting M2 polarization, and inhibiting antitumor responses of CD8^+^ T cells [89]. Pathological remodeling of the cytokine network further amplifies immune suppression, METRNL secreted by immune cells in the tumor microenvironment activates the E2F‐PPARδ pathway, leading to mitochondrial depolarization and reduced oxidative phosphorylation in CD8^+^ T cells, resulting in bioenergetic failure and functional impairment [68].

Inflammation constructs a dense immunosuppressive network through multiple mechanisms, providing a sanctuary for tumor cells to evade immune surveillance. In colorectal cancer, HMGB1 released by the inflammatory microenvironment characterized by hypoxia and glucose deprivation induces surrounding cells to secrete CXCL2, which recruits myeloid‐derived suppressor cells (MDSCs) via a CXCR2‐dependent pathway [90]. Intestinal microbial dysbiosis also contributes: *Megasphaera elsdenii* and its lipopolysaccharides activate dendritic cells (DCs) via the TLR4/NF‐κB/IRF4 pathway, inducing Th1/Th17 inflammatory responses to disrupt intestinal immune homeostasis [91]. Similar mechanisms act synergistically in other cancer types: in head and neck squamous cell carcinoma, IL‐4 activates STAT6, inducing high expression of Siglec‐G/10; this promotes TAM differentiation and immune suppression by upregulating hypoxia‐inducible factor 1 alpha (HIF‐1α) [92]; IL‐11 secreted by glioblastoma cells activates the STAT3 signal in astrocytes, inducing them to express TRAIL and trigger T cell apoptosis [93]; In brain tumors, infiltration of TREM2^+^/TIM3^+^ suppressive myeloid cells, together with blood–brain barrier dysfunction, exacerbates neuroinflammation and immune escape [94].

Inflammation‐driven abnormal communication between immune cells and tissue microenvironment‐specific regulation further consolidate immune tolerance. Dysfunction of CD4^+^ T cells plays a key regulatory role: IL‐17A secreted by them acts as a “wake‐up signal” for lung‐disseminated cancer cells, breaking metastatic dormancy [95]; Phenotypic changes in T cells themselves also contribute to immune dysfunction: in CAC, branched N‐glycans on T cells gradually increase with inflammatory progression; by endowing T cells with suppressive properties, this excludes effective antitumor immunity [96]; The STUB1–CHIC2 complex in CD8^+^ T cells directly inhibits their antitumor activity by regulating cytokine receptor expression [97]. Additionally, tissue‐specific immune regulation abnormalities exacerbate local immune dysfunction: for example, intestinal ERβ deficiency increases macrophage infiltration and reduces T cell and NK cell infiltration; steatohepatitis reduces the liver's immune clearance of colorectal metastases by altering lymphocyte cytotoxicity and localization [98].

In the inflammation–tumor association, key mechanisms disrupt immune homeostasis to drive the “inflammation–immune imbalance–tumor” process with tight logic. High fructose intake activates mTORC1 and ROS‐TGF‐β signaling, triggering abnormal Th1/Th17 differentiation that both exacerbates inflammation and impairs immune surveillance [99]. In this inflammatory state, ATP‐activated P2 purinergic receptors in the tumor microenvironment simultaneously amplify inflammation and induce immune suppression, acting as a critical link [100]. Once immune suppression forms, it shelters tumors: in advanced salivary gland cancer, immunosuppressive macrophages dominate the microenvironment, squeezing effector T cells, with subtype‐specific defects further strengthening suppression and reducing immunotherapy response [101]; in steatohepatitis, increased liver CD8^+^T cells and NK cells show reduced cytotoxicity and infiltration, failing to clear metastases [102]. Persistent inflammation‐induced immune dysfunction accumulates, allowing potentially clearable precancerous lesions to gradually develop into malignant tumors.

### 3.2 Stromal Fibrosis

Chronic inflammation induces phenotypic transformation of stromal cells such as fibroblasts via activating signaling pathways, initiating stromal fibrosis [103, 104, 105]; Stromal fibrosis, in turn, exacerbates inflammation and disrupts homeostasis, forming a pathological loop of “inflammation–stromal fibrosis–tumor,” with stromal cells serving as the core hub. In the tumorigenesis of colorectal cancer, sustained inflammation induced by inflammatory bowel disease (IBD) upregulates bradykinin B1 receptor (B1R), activates the extracellular matrix (ECM) pathway, drives the abnormal phenotypic transformation of fibroblasts, disrupts matrix homeostasis, and lays the foundation for CAC. Aging‐related pancreatic inflammation activates signaling pathways such as TGF and PDGF, promoting the transition of pancreatic stellate cells from a quiescent to an activated state; aging further shifts them toward a profibrotic subset, exacerbating tissue degeneration by secreting large amounts of matrix components [106]. In cancer cachexia, MIF secreted by tumors binds to the ACKR3 receptor on adipose stem cells, directionally inducing their differentiation toward proinflammatory and profibrotic phenotypes while inhibiting adipogenesis. This forms a pathological feature of concurrent adipose tissue inflammation and stromal fibrosis [107].

Stromal fibrosis is not merely a structural change but feeds back into pathological processes by constructing a proinflammatory microenvironment and disrupting tissue barriers, accelerating tumor progression. In primary sclerosing cholangitis, high Claudin‐1 expression in hepatic epithelial cells activates proinflammatory and stromal fibrosis‐related signaling pathways, exacerbating biliary inflammation, stromal fibrosis, and cholestasis. This chronic pathological state significantly increases the risk of hepatobiliary cancer, and Claudin‐1 expression levels are directly associated with patient disease stage and prognosis [108]. In gastric cancer development, the PDGFRα‐positive fibroblast subset expands significantly during the metaplasia stage, closely contacting gastric epithelial cells; signaling molecules secreted by these fibroblasts promote the transformation of metaplastic gastric cells to dysplasia, directly driving the progression of precancerous lesions [109].

Abnormal crosstalk between stromal cells is the core hub linking inflammation, stromal fibrosis, and tumor progression, with the phenotypic regulation of cancer‐associated fibroblasts (CAFs) being particularly critical. In pancreatic cancer, endogenous p38 MAPK signaling in tumor cells drives overexpression of IL‐1α by coordinating Sp1 and NF‐κB‐p65 transcription factors; this activates the proinflammatory phenotype of CAFs in a paracrine manner [110], maintaining a desmoplastic matrix and immunosuppressive microenvironment, leading to chemotherapy resistance; high CALB2 expression in both CAFs and cancer cells forms a vicious cycle: activated CAFs via CALB2 upregulate CALB2 expression in cancer cells through the IL6‐STAT3 pathway; cancer cells further promote the inflammatory phenotype of CAFs via the Ca^2^^+^–CXCL14 axis, collectively driving metastasis and immune suppression [111]. In liver pathology, intrahepatic IgA complexes bind to the CD71 receptor on CAFs, inducing their polarization into a stromal phenotype with high PD‐L1 expression; coculture with CD8^+^ T cells significantly inhibits their cytotoxic function, highlighting the mediating role of CAFs in immune escape [112].

Targeted intervention of stromal cell phenotypes and signaling pathways has become a potential strategy to break the inflammation–stromal fibrosis–tumor pathological cycle. In pancreatic cancer treatment, combined MEK and STAT3 inhibition reduces the polarization of proinflammatory CAFs while enriching CAF subsets with mesenchymal stem cell‐like characteristics. This is accompanied by M2‐to‐M1 reprogramming of TAMs and enhanced CD8^+^ T cell recruitment; combination with programmed death 1 (PD‐1) inhibitors significantly improves clinical benefits for patients [113]; Inhibiting p38 MAPK reduces IL‐1α secretion by tumor cells, weakens the inflammatory phenotype of CAFs, and enhances chemotherapy sensitivity. In the prevention and treatment of breast cancer lung metastasis, targeted knockout of the Ptgs2 gene (encoding COX‐2) in lung fibroblasts reverses the immunosuppressive microenvironment mediated by their secreted PGE_2_, reduces premetastatic niche formation, and enhances the antimetastatic activity of PD‐1 blockade therapy [114]. In liver diseases, small extracellular vesicles derived from engineered mesenchymal stem cells deliver USP10, reprogramming macrophage phenotypes via the KLF4‐NF‐κB/STAT6 pathway. This effectively alleviates fibrosis, providing new insights for blocking the transformation of liver disease to liver cancer [115]. Mechanistic insights into the inflammation–stromal fibrosis–tumor axis highlight the critical role of stromal cells in tumor progression. Current strategies targeting stromal cell phenotypes or signaling pathways have shown potential; further exploration in the future will provide important directions for breaking the pathological loop and developing new tumor therapies.

### 3.3 Microbial Dysbiosis

Chronic inflammation directly triggers abnormal remodeling of the structure and function of intestinal and mucosal microbial communities by disrupting host immune defense and microenvironmental balance. In colitis, persistent intestinal inflammation impairs the immune function of Peyer's patches, enabling ectopic translocation of *Alcaligenes faecalis* (a resident bacterium). This bacterium not only inhibits the function of immune cells such as B cells and DC cells to reduce IgA^+^ B cell homing but also induces vinculin acetylation by secreting acetic acid, disrupting intestinal barrier integrity. This forms an initial dysregulation cycle of “inflammation–microbial translocation–barrier disruption” [116]. Inflammation associated with innate immune deficiency also drives microbial dysbiosis: mice with myeloid cell‐specific TRAF3 deficiency exhibit intestinal microbial disorders and migration of commensal bacteria to the liver due to impaired innate immune function. Antibiotic clearance of commensal bacteria effectively prevents the occurrence of B cell lymphoma, confirming that microbial dysbiosis caused by the synergy of inflammation and immune deficiency is a key trigger for tumorigenesis [117]. Additionally, the chronic inflammatory state of Crohn's disease creates conditions for intestinal colonization of the oral commensal bacterium *Veillonella*; its secreted proinflammatory lipopolysaccharides activate the c‐Jun/c‐Fos signal to inhibit the expression of the bile acid transporter ASBT, exacerbating intestinal microenvironmental disorder [118]. The direct immunomodulatory effect of viral proteins exacerbates tolerance: the human cytomegalovirus IE1 protein downregulates Rb/p53 and upregulates Myc/EZH2, inducing the formation of polyploid giant cancer cells and enhancing tumor stemness while evading immune clearance [119].

Microbial dysbiosis is not merely a disorder of microbial communities; it further exacerbates inflammatory responses through the release of metabolites and the action of virulence factors, providing a pathological basis for tumorigenesis. *Fusobacterium nucleatum* is enriched in colorectal cancer; by disrupting communication between TAMs and IgA plasma cells, it inhibits the production of secretory IgA, leading to impaired mucosal immunity and increased bacterial infiltration. This further worsens tumor prognosis through chronic inflammation [120]. Virulence factors CagA and VacA of *Helicobacter pylori* directly induce chronic gastritis; when the anti‐inflammatory effect of host mucin MUC1 is insufficient, this activates the NLRP3 inflammasome to increase IL‐1β secretion, promoting the transformation of gastric mucosa to dysplasia and adenocarcinoma [121, 122]. Microbial dysbiosis caused by viral infection also amplifies inflammatory effects: hepatitis B virus (HBV) does not cause cancer alone, but enhances chemically induced liver inflammation by regulating the IL‐33/regulatory T cell axis, synergistically promoting hepatocellular carcinoma development [123]; Epstein–Barr virus (EBV) establishes latent infection to express low‐immunogenicity products, triggering chronic inflammation while helping tumor cells evade immune surveillance [122, 124].

Structural imbalance and functional abnormalities of the microbiota accelerate tumor progression directly or indirectly through multiple mechanisms. Biofilm formation by the intestinal microbiota is an important protumor link: microbial aggregates in the tumor microenvironment form a “protective shield” via extracellular polymeric substance matrices, resisting host immune attacks and releasing bioactive molecules to exacerbate chronic inflammation and immune evasion. Stage‐dependent enrichment of specific microbiota is closely associated with tumor progression: *Alcaligenes faecalis* increases in a stage‐dependent manner in colitis, adenomas, and colorectal cancer, with its barrier‐damaging effect on the intestine gradually strengthening as the disease progresses [116]; Additionally, microbiota‐mediated abnormal activation of signaling pathways drives tumor cell proliferation: microbial dysbiosis caused by epithelial Regnase‐1 deficiency activates the IL‐17/NFKBIZ/ERK signaling pathway; sophoramine can inhibit MAPK‐mediated inflammation by restoring the intestinal microbiota, which conversely confirms the regulatory role of the microbiota in tumor‐related signaling pathways [125, 126]. Taking impaired immune defense and microenvironmental imbalance as breakthrough points, chronic inflammation initiates structural and functional abnormalities of the microbiota. This initial microbial dysbiosis not only exacerbates local inflammatory responses but also forms a pathological cycle through multiple pathways such as metabolic disorder, barrier disruption, and immune escape, providing a critical microecological basis for tumor occurrence and development.

### 3.4 Vascular Neoplasia

A tight pathological loop exists between chronic inflammation, vascular neoplasia, and tumors: inflammation drives abnormal neovascularization via activating signaling pathways; disordered new blood vessels, in turn, provide conditions for inflammation amplification and tumor growth. The three mutually reinforce each other, forming a protumor cycle [127, 128, 129]. By activating specific signaling pathways, chronic inflammation serves as the core initiating factor driving vascular neoplasia, laying a vascular foundation for tumorigenesis. In IBD, inflammatory signals activate the STAT1 pathway, upregulating the expression of transglutaminase 2 (TGM2); TGM2 interacts with vascular endothelial growth factor receptor 2 (VEGFR2) and promotes its phosphorylation, directly driving inflammation‐associated angiogenesis and accelerating the transformation of colitis to cancer [130]. The chemokine CXCL16–CXCR6 axis exhibits pleiotropic regulatory effects: its soluble form (sCXCL16) not only enhances tumor cell migration but also remodels the tumor vascular network through direct proangiogenic effects; sustained activation of its transmembrane form (mCXCL16) may induce immune escape, further linking the pathological chain of inflammation, vascular neoplasia, and tumor progression [131]. In prostate cancer, *IL‐30* secreted by tumor cells maintains its own expression via an autocrine loop, while activating multiple signaling pathways such as Src and STAT3 to promote endothelial cell proliferation and vascular sprouting. By upregulating the expression of endothelial cell immune regulatory genes and oncogenes, it simultaneously drives inflammation amplification and tumor progression [132].

Abnormal neovascularization is not merely a nutritional channel; its structural disorganization and abnormal signal transduction functions can further act on inflammatory responses and tumor progression, exacerbating the pathological state of the tumor microenvironment. Such blood vessels usually exhibit significantly increased permeability: on the one hand, they provide sufficient oxygen and nutrient supply for tumor cells to meet their malignant proliferation needs; on the other hand, they can promote the extensive diffusion of inflammatory factors and create favorable conditions for the invasion and metastasis of tumor cells [133]. Pericytes, as key components of the vascular niche, also amplify pathological effects through dysfunction: loss of soluble guanylate cyclase (sGC) in pericytes disrupts endothelial cell‐pericyte communication, perturbing the phenotypes of CAFs and TAMs via paracrine signals. Although this inhibits tumor growth, it conversely confirms the regulatory role of vascular components in the microenvironment [134]. Inhibition of MCL‐1 in breast cancer‐associated fibroblasts (bCAFs) induces their transition to a proinflammatory, proangiogenic phenotype; by secreting VEGF to enhance the tube‐forming ability of endothelial cells, this further consolidates the pathological cycle [135].

Targeted intervention of the inflammation–vascular neoplasia axis has become a potential therapeutic direction to break the protumor cycle, with a focus on signaling pathway and vascular component regulation. At the level of angiogenic signals, the compound 2‐Desaza‐annomontine (C81) reduces VEGFR2 expression and downstream signal activation by inhibiting CDC2‐like kinases and the WNT/β‐catenin pathway; it also inhibits endothelial inflammation, achieving dual blockade of angiogenesis and inflammation [136]; Ru(II) complexes simultaneously block inflammatory transcription and angiogenic signals by inhibiting NF‐κB nuclear translocation and VEGFR2 phosphorylation, showing activity in triple‐negative breast cancer [137]. At the level of the vascular microenvironment, VEGF‐targeted drugs (e.g., sunitinib) can reverse the anergic state of tumor endothelial cells, enhancing immune cell infiltration by upregulating ICAM‐1, combining antiangiogenesis with immune activation [138]; Targeted inhibition of pericyte sGC can increase the sensitivity of tumor blood vessels to conventional antiangiogenic therapy, providing new ideas for combination therapy. Additionally, early intervention of stromal PPARγ activity maintains vascular endothelial stability and inhibits pericyte transition to a protumor phenotype, significantly delaying tumorigenesis. This suggests an important impact of intervention timing on therapeutic efficacy [139]. This highlights the core hub role of vascular neoplasia in pathological processes. Current strategies targeting signaling pathways or the vascular microenvironment have shown potential; further exploration in the future will provide key directions for breaking the pathological loop and developing new tumor therapies.

### 3.5 Neurotoxicity

Chronic inflammation can activate neurotransmitter pathways, neuropeptide signals, and neuroimmune circuits, induce abnormalities in the neuroregulatory system, and lay a pathological foundation for tumorigenesis and development. In CAC, inflammatory signals upregulate ALKAL2 expression in TRPV1^+^ sensory neurons; as an ALK receptor ligand, ALKAL2 activates ALK signaling in the colonic mucosa. Activating TRPV1^+^ neurons directly exacerbates tumor growth, while silencing these neurons significantly inhibits tumor progression. This reveals the core mechanism by which sensory nerves drive tumors via inflammation‐regulated signaling pathways [140]. During colorectal cancer progression, there is a transitional loss of local sympathetic nerve input; this neurotoxicity disrupts the balance of norepinephrine‐mediated regulation of cancer cell chemokine expression via α2‐adrenergic receptors, leading to reduced CD8^+^ T cell recruitment and increased immunosuppressiveness of the tumor microenvironment. This forms an initial protumor cycle of “inflammation–neurotoxicity–immune suppression” [141]. Additionally, inflammation‐associated neurotoxicity contributes to pathological processes: tumor inflammation‐associated neurotoxicity (TIAN) occurring in patients with central nervous system lymphoma after immunotherapy is characterized by dense macrophage infiltration and myelin loss; its lesions are closely associated with tumor foci, reflecting the synergistic effect of inflammation and nerve damage in the tumor microenvironment [142].

Sustained abnormalities in the neural regulatory system form a malignant interaction with tumors by remodeling the microenvironment and amplifying inflammatory signals. In cancer cachexia, inflammation‐driven neuroimmune circuits play a key role: elevated IL‐6 during systemic inflammation is sensed by brain stem cell factor‐sensing circuits; via the basal ganglia, this is converted to reduced mesolimbic dopamine, triggering behavioral symptoms such as apathy. This neural signal disorder further impairs the body's ability to cope with disease [143]; Simultaneously, cancer‐induced systemic inflammation alters vagal tone, disrupting the brain–liver vagal axis and leading to depletion of the hepatic transcriptional regulator HNF4α. This causes hepatic metabolic reprogramming and exacerbates systemic inflammation, conversely promoting cachexia and tumor progression [144]. Abnormal activation of neural signaling pathways also directly regulates tumor biological behavior: the CXCL13/CXCR5 chemokine pathway is significantly upregulated in inflammation and tumor‐associated pain; by activating signals such as p38 MAPK and NF‐κB, it enhances neuronal excitability and promotes neuroinflammation. This neural signal disorder not only exacerbates patient suffering but also provides a favorable microenvironment for tumor growth through the amplifying effect of immune cell infiltration and inflammatory factor release, highlighting the pathological hub role of abnormal neural regulation between inflammation and tumors [145]. Taking the abnormal activation of neurotransmitters, neuropeptides, and neuroimmune circuits as entry points, chronic inflammation induces dysfunction of the neural regulatory system. This initial neurotoxic change not only disrupts the homeostatic balance of the neuro–immune–tumor axis but also forms a pathological cycle through signal crosstalk and microenvironment remodeling, becoming an important pathological link connecting inflammation and tumor progression.

**FIGURE 2:**
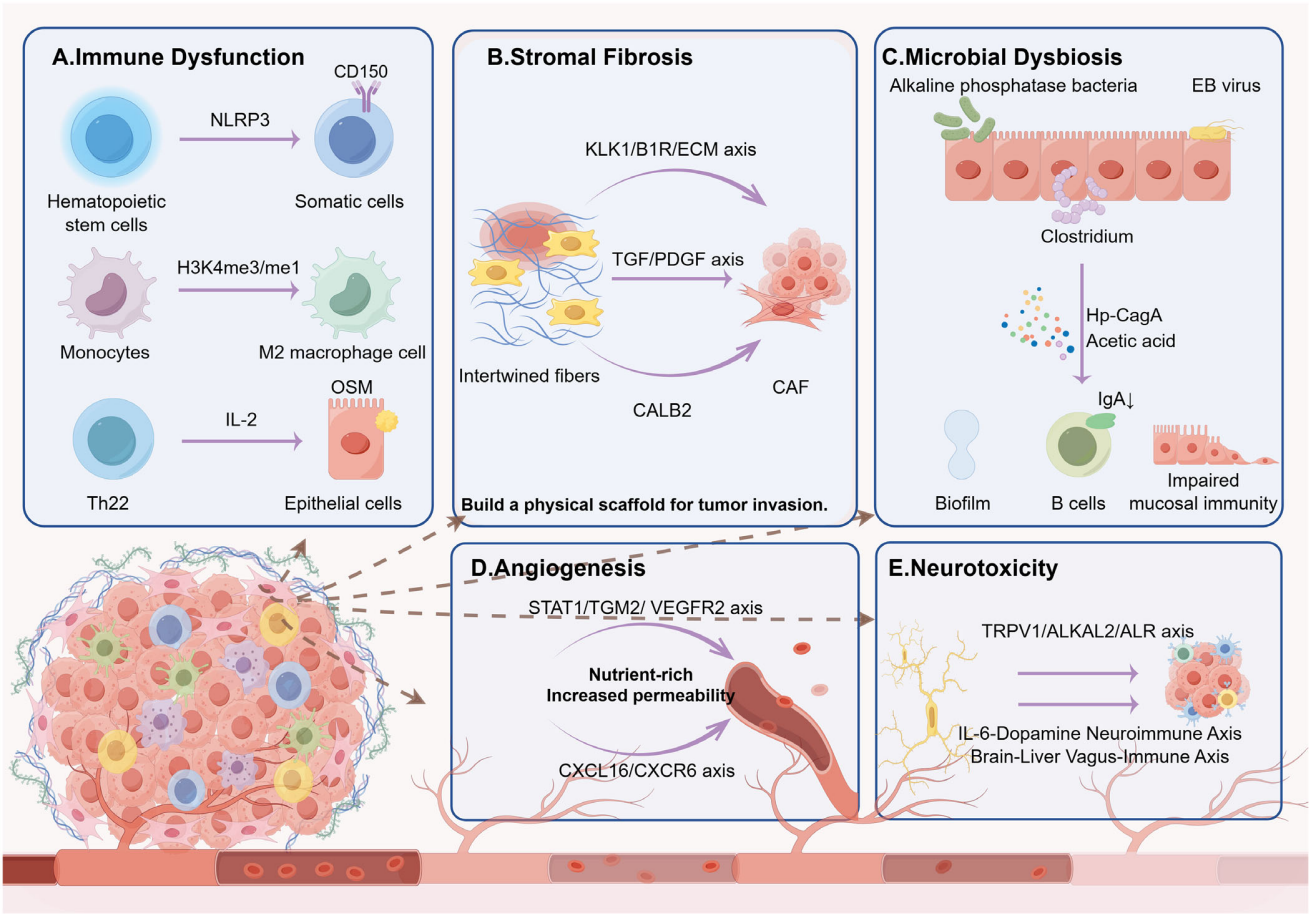
Extrinsic stimuli of inflammation‐to‐cancer transition. Core components: Immune dysfunction, stromal fibrosis, microbial dysbiosis, vascular neoplasia, and neurotoxicity form a protumor synergistic regulatory network centered on the tumor microenvironment.

In summary, immune dysfunction, stromal fibrosis, microbial dysbiosis, vascular neoplasia, and neurotoxicity collectively constitute the core external attractions of inflammation‐to‐tumor transformation, as shown in Figure 2. With the tumor microenvironment as the hub, they form a synergistic regulatory network: immune dysfunction removes immune constraints on tumor growth; stromal fibrosis and vascular neoplasia provide structural support and material guarantees, respectively; microbial dysbiosis continuously amplifies inflammatory signals; and neurotoxicity exacerbates microenvironmental disorder via the neuro–immune–tumor axis. These external attractions correspond to the internal drivers of inflammation‐induced tumorigenesis, collectively shifting inflammation from physiological repair to pathological tumorigenesis. Clarifying the mechanisms and interactive logic of these external attractions can provide novel intervention ideas and therapeutic targets for targeting the tumor microenvironment and blocking the inflammation‐induced tumorigenic process.

## 4 Therapeutic Targets and Drug Development for Inflammation‐Related Tumors

Targeting inflammation‐related pathways and optimizing diagnosis and treatment strategies have become core directions in drug development for inflammation‐related tumors, addressing the mechanisms of inflammation‐driven tumorigenesis, progression, and therapeutic resistance. From preclinical target discovery and validation, to the screening and application of auxiliary diagnostic factors, and further to the design of combined treatment regimens and the development of novel delivery systems, multidimensional research collectively drives the transition of inflammation‐related tumor treatment from basic mechanisms to clinical translation, opening new possibilities for improving patient prognosis.

### 4.1 Clinical Research

Currently, the association between inflammation and tumors has entered a critical stage of clinical translation from basic mechanism exploration, emerging as one of the most promising research directions in oncology. Clinical studies have clearly outlined the complete chain by which inflammation initiates malignant cellular transformation via “internal drives” (e.g., genomic disorder, epigenetic memory) and then shapes a protumor microenvironment through “external attractions” (e.g., immune dysfunction, vascular neoplasia). Translational research is gradually converting these mechanistic findings into the basis for clinical intervention as shown in Table 2. However, current clinical research still exhibits the characteristic of “clear mechanisms coexisting with translational bottlenecks”:

**TABLE 2:** Clinical studies on internal/external drivers of inflammation–tumor transformation.

Category	Subcategory	Clinical trial no.	Trial phase	Cancer type	Inflammation–tumor molecular mechanism	Key data	References
Internal drives	Genomic disorder	NCT02223923	Phase I	Advanced solid tumors	Inflammation activates DNA damage pathways; ARID1A‐deficient tumors have high inflammation levels; ATR inhibitors target repair defects	ORR 8%, DCR 60%; Genomically defective + highly inflammatory tumors show durable responses	[146]
		None (basic research)	None	Prostate cancer	Oncogenic RAS + p53 deletion exacerbates DNA damage, induces SASP to release IL‐6/IL‐8	SASP induces EMT; increased invasiveness	[147]
	Epigenetic memory	NCT03542266	Phase II	Peripheral T‐cell lymphoma (PTCL)	Inflammation‐related TET2 mutations cause chemotherapy resistance; azacitidine upregulates inflammatory genes to reprogram the microenvironment	CR rate 75% (88.2% in PTCL‐TFH subtype), 2‐year OS 68.4%; OS prolonged in TET2‐mutated patients (*p* = 0.015)	[148]
		NCT02476617	Phase II	Chronic infection‐associated tumors	Inflammation induces CD8+T cell exhaustion; TOX/HIF1A forms epigenetic scars	Epigenetic scars persist after infection cure; T cell dysfunction not reversed	[149]
		NCT02905188, NCT02932956	Phase I	GPC3‐positive solid tumors	IL‐15‐modified chimeric antigen receptor T‐cell (CAR‐T) inhibits SWI/SNF, activates type I interferon inflammatory signals	DCR 66%, ORR 33%	[150]
	Mitochondrial stress	NCT04691765	Phase I	Chronic lymphocytic leukemia	Inflammation activates NF‐κB pathway; mitochondrial oxidative stress imbalance; anakinra inhibits NF‐κB but upregulates IFN signals	100% lymph node reduction within 1 month; IFN signal upregulation leads to loss of response	[151]
	Metabolic reprogramming	ChiCTR2100053521	Phase III	Metastatic colorectal cancer	Inflammation drives elevated LDH; Quyu Jiedu Decoction downregulates inflammatory factors to improve metabolism	3‐year DFS 47.4% (32.4% in control group, HR = 0.515); Inflammatory markers and LDH decreased (*p* < 0.05)	[152]
		NCT03409848	Phase II	HER2‐positive esophagogastric adenocarcinoma	Inflammatory IL‐6 elevation causes metabolic disorders; neutrophil/lymphocyte ratio associated with immune resistance	Nonchemotherapy regimen showed better OS in low‐inflammation patients; neutrophil/lymphocyte ratio correlated with survival	[153]
		NCT01387269, NCT01387282	Phase III	Non–small‐cell lung cancer (cachexia)	Systemic inflammation (mGPS ≥ 1) exacerbates metabolic disorders; anamorelin improves inflammation‐related cachexia	Patients with mGPS = 2 had weight gain > 5%; grip strength and FAACT A/CS scores improved (*p* < 0.001)	[154]
External attractions	Immune dysfunction	EudraCT‐No.2021‐002073‐26	Phase Ib/II	Melanoma	Inflammation drives IFN‐γ/CXCL10 secretion; CD14+ monocytes mediate CAR‐T‐related immune dysfunction	IFN‐γ elevated in patients with breakthrough toxicity; IL‐4/IL‐10 negatively correlated with toxicity (*p* < 0.05)	[155]
		NCT05013450	Phase Ib/II	Non–small‐cell lung cancer	Inflammation drives activation of IL‐4 signaling axis; regulates myeloid‐derived suppressor cell production	1/6 patients achieved near‐complete response; circulating monocytes decreased; CD8+T cell infiltration increased	[156]
		ChiCTR2000032339	Phase II	Tumor patients with sepsis	Sepsis inflammation exacerbates immunosuppression; carrimycin upregulates HLA‐DR and CD8+T cell levels	HLA‐DR increased (*p* = 0.023); CD8+T cells increased (*p* < 0.035); SOFA and APACHE II scores decreased	[157]
		NCT03063450	Phase III	Malignant mesothelioma	Inflammation drives tertiary lymphoid structure formation; EMT‐related inflammatory signals cause immune resistance	Nivolumab ORR 10.3%; IL24/CCL19 upregulated in responders	[158]
		None (Phase II trial)	Phase II	Non–small‐cell lung cancer	Inflammation drives immunosuppression; JAK1 inhibitors block inflammatory signals to improve CD8+T cell differentiation	Itacitinib combined with PD‐1 blockade: ORR 67%; CD8+T cell effector function enhanced	[159]
		NCT02706405	Phase I	Diffuse large B‐cell lymphoma	Inflammation induces elevated sPD‐L1; inhibits CAR‐T function; PD‐L1 blockade timing affects inflammation‐immune balance	Higher CAR‐T expansion rate in durvalumab postinfusion group; 45% late regression rate; sPD‐L1 negatively correlated with efficacy	[160]
		NCT03056339	Phase I/II	CD19+B‐Cell malignancies	Inflammation/hypoxia regulates umbilical cord blood CAR‐NK function; high‐quality donor NK cells have low‐inflammatory stress programs	ORR 48.6%, 1‐year OS 68%; High‐quality CBU‐derived CAR‐NK showed better activity	[161]
	Stromal fibrosis	None (intervention trial)	None	Rectal cancer	Radiotherapy induces inflammatory factor release to promote stromal fibrosis; synbiotics inhibit inflammation to reduce fibrosis	Inflammation and fibrosis reduced; bacterial diversity retention rate higher than control group	[162]
		None (intervention trial)	None	PDAC	TGFβ inhibition induces fibroblast inflammatory phenotype; secretion of autocrine chemokines promotes fibrosis and resistance	IOA‐289 synergizes with galunisertib to restore gemcitabine sensitivity; prolonged survival in mice	[163]
		NCT02843425	None	Patients with colorectal tumor history	Prebiotics regulate intestinal microbiota; modulate inflammation and stromal fibrosis via FGF‐19	FGF‐19 increased (*p* = 0.01); intestinal inflammatory markers decreased	[164]
	Microbial dysbiosis	NCT03654833	Phase II	Malignant mesothelioma	Microbial dysbiosis enhances CD68+ monocyte inflammatory infiltration; affects PD‐L1/VEGF blockade efficacy	ORR higher in patients with normal microbial ratio than in dysregulated patients (*p* < 0.01); positively correlated with CD8+T cell infiltration	[165]
		None (observational study)	None	Gastric cancer	Oral bacterial clusters enriched after *Helicobacter pylori* eradication; maintain gastric mucosal chronic inflammation to promote precancerous lesions	Roseburia/Sphingomonas enriched in patients with persistent inflammation; 78% detection rate of oral bacterial clusters in patients with atrophy/IM (22% in control group)	[166]
	Vascular neoplasia	None (Phase IB trial)	Phase IB	Inflammatory breast cancer	Inflammation drives high VEGF expression to promote vascular neoplasia; SU5416 targets VEGFR	Tumor blood flow decreased (*p* = 0.033); trend of reduced microvessel density	[167]
		None (intervention trial)	None	Metastatic melanoma	VEGF mediates inflammatory immunosuppression; bevacizumab combined with ipilimumab modulates vascular neoplasia and inflammation balance	DCR 67.4%, median OS 25.1 months; CD8+T cell infiltration increased	[168]
		NCT02495922	Phase III	Multiple myeloma	Anti‐inflammatory effect of lenalidomide inhibits VEGF; combined therapy modulates vascular microenvironment	2‐year PFS 65.8% in combined therapy group; VEGF decreased	[169]
	Neurotoxicity	NCT04099797	Phase I	Pediatric central nervous system tumors	CAR‐T activates inflammatory pathways; elevated IL‐6/IP‐10 mediates neurotoxicity	88% of patients developed grade 1 neurotoxicity; 100% control rate with anakinra; 29% achieved partial response	[170]
		NCT04150913	None	Large‐cell lymphoma	Inflammation drives activation of IFN‐γ pathway; mediates CAR‐T‐related neurotoxicity and immune dysfunction	IFN‐γ level increased 3.1‐fold in patients with neurotoxicity; CXCL10 expression upregulated 2.8‐fold	[171]

Data sources—Clinical trial registration website: ClinicalTrials.gov (international clinical trial registry, https://clinicaltrials.gov/, accessed on March 3, 2025).

On one hand, drugs targeting inflammatory pathways such as IL‐1 and NF‐κB have shown activity in combined treatments for cancers including chronic lymphocytic leukemia [151] and pancreatic cancer [163]. For example, anakinra can balance inflammatory signals by regulating mitochondrial stress, and the response rate of JAK inhibitors combined with anti‐PD‐1 therapy reaches 67% in non–small‐cell lung cancer [159]; On the other hand, the “dual nature” of inflammation poses significant challenges: acute inflammation can activate antitumor immunity, while chronic inflammation promotes tumorigenesis. Some patients even experience hyperprogressive disease due to peritumoral pathogenic inflammation during immunotherapy, and long‐term use of anti‐inflammatory drugs may be accompanied by infection risks or cardiovascular toxicity. Additionally, individual heterogeneity in clinical benefits is prominent, and there is a lack of universal inflammatory markers to guide precise medication.

The above research evidence systematically demonstrates the translational trajectory of inflammation–tumor research from basic science to clinical practice, and also reveals the core breakthroughs and unsolved problems in the current field. At the mechanism level, the application of single‐cell sequencing and spatial transcriptomics will further clarify the subtype classification of the tumor inflammatory microenvironment and identify regulatory targets for key pathological links. In terms of treatment strategies, combined regimens of “inflammatory pathway blockade + immune activation” will become more mature—for example, IL‐1β inhibitors combined with PD‐1 inhibitors to improve the immunosuppressive microenvironment—while combined intervention targeting compensatory signaling pathways can overcome single‐drug resistance [160]. Additionally, personalized medicine will become a core direction: patient stratification via inflammation‐related scores such as modified Glasgow Prognostic Score (mGPS) [172], combined with nontraditional approaches such as intestinal microbiota regulation and epigenetic modulators, to build a complete system of “risk prediction–precision intervention–efficacy monitoring.” With the clinical validation of these strategies, inflammation‐targeted therapy is expected to evolve from an auxiliary approach to a core pillar of comprehensive tumor treatment, opening new pathways for improving patient survival benefits.

### 4.2 Auxiliary Diagnostic Factors

The pathological association between inflammation and tumors runs through the entire process of disease occurrence, progression, and outcome. As molecular “mirrors” of their interaction, inflammation‐related markers have become core bases for tumor diagnosis, treatment selection, prognosis assessment, and recurrence monitoring. Ranging from cellular ratios and protein molecules in blood to genetic characteristics and imaging phenotypes in tissues, these markers accurately capture inflammation‐driven pathological changes in tumors, providing objective and quantifiable references for clinical diagnosis and treatment, and promoting the transition of tumor care from empirical medicine to precision medicine [173, 174, 175]. Inflammation‐related molecular and cellular markers can effectively identify high‐risk populations for tumors, providing noninvasive detection tools for early diagnosis. In colorectal cancer, elevated neutrophil‐to‐lymphocyte ratio (NLR) and decreased lymphocyte‐to‐monocyte ratio (LMR) are significantly associated with increased disease risk: the risk in the highest NLR quartile is 1.14 times higher than that in the lowest quartile, and the risk in the lowest LMR quartile is 49.7% lower than that in the highest quartile. Both can serve as practical biomarkers for early colorectal cancer screening [176]. In pancreatic diseases, the tracer V‐1520 shows specific accumulation in pancreatic cancer‐related inflammation, with outstanding ability to identify early high‐risk precancerous lesions, facilitating precise surgical resection to reduce recurrence risk [177]. In patients with alcohol‐related hepatitis, the SASP factor growth differentiation factor 15 (GDF15)—associated with hepatocellular senescence—is highly expressed in plasma. It not only reflects the degree of liver inflammation but also is closely associated with patient mortality risk, providing a reference for subsequent tumor risk assessment. By capturing the early association between inflammation and tumorigenesis, these markers enable early disease identification and risk stratification [178].

Inflammation‐related indicators can accurately reflect the state of the tumor microenvironment, providing dynamic bases for treatment strategy formulation and efficacy monitoring. In head and neck squamous cell carcinoma, elevated inflammatory markers (IL‐6, sCD25, sTIM‐3) and increased levels of peripheral blood neutrophils and cell‐free DNA predict poor response to PD‐1 inhibitor treatment, while patients lacking these high‐risk markers derive significant benefits from pembrolizumab monotherapy [179]. In advanced non–small‐cell lung cancer patients receiving chemoimmunotherapy or immunotherapy, the early kinetic characteristics of serum amyloid A (SAA) have clear predictive value: patients with a “flare response” (initial increase followed by decrease) have significantly prolonged median progression‐free survival—29.8 months in the chemoimmunotherapy group and 19.9 months in the immunotherapy group—far exceeding the 7.4 months and 2.1 months in nonresponder groups [180]. In non‐Hodgkin lymphoma, the InflaMix model—integrating 14 inflammation and organ function indicators—effectively identifies patients at high risk of CAR‐T treatment failure, with a hazard ratio of 2.98. It maintains accuracy even when only six routine laboratory indicators are retained, providing support for treatment regimen optimization [181].

Inflammation‐related molecular, cellular, and imaging markers can comprehensively assess tumor progression risk, providing core bases for prognostic stratification. In colorectal cancer, the MALMPS model—constructed based on metabolic genes—reveals that the high‐risk subgroup has significantly worse prognosis than the low‐risk subgroup due to inflammation activation and abnormal carbohydrate/lipid metabolism. The predictive efficacy of this model is superior to traditional clinical and molecular characteristics [182]. In multiple myeloma patients before CAR‐T treatment, bone‐independent extramedullary disease (EMD) and high metabolic tumor volume (MTV) detected by positron emission tomography/computed tomography (PET/CT) both indicate poor prognosis: the median progression‐free survival of EMD patients is only 3 months, while patients with metabolic complete remission after treatment have significantly improved prognosis [183]. A pan‐cancer study shows that in patient clusters integrated with nutritional and inflammatory characteristics, the “severely impaired/inflamed” group has a 2.96‐fold higher mortality risk than the “fit” group. Moreover, the impact of inflammatory characteristics on prognosis is independent of tumor origin and metastatic status, highlighting their value as universal prognostic indicators [184].

Dynamic changes in inflammation‐related indicators can promptly capture tumor recurrence signals, providing early warnings for intervention timing selection. Multiomics analysis of solitary fibrous tumors of the central nervous system shows that the inflammatory molecular subtype—regulated by both hypoxia and inflammation—has significantly higher recurrence and metastasis risks than other subtypes. FER kinase inhibitors can reduce recurrence risk by inhibiting inflammation‐related proliferation signals [185]. In colorectal cancer patients after treatment, the recurrence risk of the high‐risk subgroup in the MALMPS model continues to increase, and its inflammation activation characteristics are associated with abnormalities in the IGF‐1R and Wnt/β‐catenin pathways, providing clues for selecting recurrence intervention targets [182]. In non‐Hodgkin lymphoma patients after CAR‐T treatment, those with persistent high‐inflammatory characteristics indicated by the InflaMix model have significantly higher recurrence rates, and dynamic monitoring of this model's indicators can detect disease progression earlier than imaging [181]. These findings confirm that inflammatory markers can serve as “dynamic scales” for tumor recurrence monitoring, facilitating precise intervention. In summary, inflammation‐related markers play key roles throughout the entire tumor diagnosis and treatment cycle, from early screening to recurrence monitoring. With technological advancements, their clinical value will be further highlighted, providing solid support for personalized diagnosis and treatment.

### 4.3 Combined Treatment Regimens

Inflammation is not only a core driver of inflammation‐to‐tumor transformation but also plays a key role in tumor therapeutic resistance. Whether it is immunotherapy, chemotherapy, radiotherapy, or surgery, chronic or treatment‐related acute inflammation can weaken therapeutic efficacy and induce chemoresistance by remodeling the tumor microenvironment, activating abnormal signaling pathways, and sheltering residual tumor cells [186]. Deciphering the unique mechanisms by which inflammation induces resistance in different treatment modalities is key to breaking through clinical treatment bottlenecks. Abnormal activation of inflammatory cells, overactivation of inflammatory pathways, and abnormal secretion of inflammatory factors collectively constitute the key microenvironmental basis for tumor progression. Targeted anti‐inflammatory agents addressing these core elements, when combined with clinical modalities such as chemotherapy, immunotherapy, and radiotherapy, can enhance efficacy and reduce toxicity by precisely regulating inflammatory responses, emerging as an important direction in tumor treatment as shown in Table 3.

**TABLE 3:** Combination therapy regimens targeting inflammatory components.

Inflammatory target category	Specific target	Cancer type	Mechanism	Targeted intervention strategy	References
Inflammatory cells	Treg cells	NAFLD‐related hepatocellular carcinoma (immunotherapy)	Inflammation‐mediated TNFSF14‐TNFRSF14 activation promotes Treg‐CAF immunosuppressive niche formation, leading to anti‐PD‐1 resistance.	Target Treg‐CAF interaction to disrupt the immunosuppressive niche and reverse anti‐PD‐1 resistance.	[187]
	CAFs	NAFLD‐related hepatocellular carcinoma (immunotherapy)	Treg‐CAF immunosuppressive niche formation mediates anti‐PD‐1 resistance.	Target Treg‐CAF interaction to disrupt the immunosuppressive niche.	[187]
	TH17 cells	ER‐negative breast cancer (immunotherapy)	Th17 cells inhibit epithelial chemokine expression via C/EBPβ, impeding immune infiltration and forming “immune desert” (reduced immunotherapy sensitivity).	Regulate Th17 function to abrogate chemokine suppression and reshape tumor immune microenvironment.	[188]
	MDSCs	Castration‐resistant prostate cancer (targeted therapy)	Inflammation‐associated PTEN deletion/CXCR2 overexpression recruits MDSCs, forming immunosuppressive microenvironment and driving enzalutamide resistance.	Combine PTEN restoration and CXCR2 depletion to reduce MDSC recruitment and reshape antitumor immunity.	[189]
	Neutrophils	Breast cancer, pan‐cancer	Inflammation reprograms bone marrow granulopoiesis, promoting neutrophil immunosuppressive phenotype transformation and tumor metastasis.	Anti‐IL‐1β therapy reverses granulopoiesis reprogramming, restores neutrophil function, and reduces metastasis risk.	[190]
	Macrophages	Lung tumors (immunotherapy)	Acute inflammation drives macrophage tumor‐killing phenotype; chronic inflammation induces macrophage protumor phenotype (promoting tumor progression).	Target macrophage inflammatory characteristics to reprogram antitumor function.	[191]
Inflammatory factors	IL‐2	Pan‐cancer (immunotherapy)	Inflammation‐driven abnormal IL‐2 secretion causes immune activation with vascular leak syndrome (limiting clinical application).	IL‐2/IL‐10 fusion (DK210) retains immune activation, restricts Treg expansion, and decouples immune activation from toxicity.	[192]
	IL‐1β	Breast cancer	Tumor‐associated inflammation via IL‐1βreprograms bone marrow granulopoiesis, promoting neutrophil immunosuppressive phenotype and metastasis.	Anti‐IL‐1β therapy reverses granulopoiesis reprogramming and restores neutrophil function.	[190]
	CXCL10	Pan‐cancer (chemotherapy)	Abnormal TNFα signaling in inflammatory microenvironment induces high CXCL10 expression, causing vascular leakage and impairing nanoparticle‐encapsulated chemotherapeutic delivery.	CXCL10‐neutralizing antibodies optimize drug delivery; exogenous CXCL10 compensates delivery defects in low‐pericyte tumors.	[193]
	TNFα	Pan‐cancer (chemotherapy)	Inflammation‐driven excessive TNF‐α secretion induces vascular disorder/permeability and upregulates CXCL10, exacerbating chemotherapeutic delivery barriers.	Target CXCL10 to indirectly intervene in TNFα‐associated vascular leakage.	[193]
	TNFSF14	NAFLD‐related hepatocellular carcinoma (immunotherapy)	Inflammation‐mediated TNFSF14‐TNFRSF14 activation promotes Treg‐CAF immunosuppressive niche formation, leading to anti‐PD‐1 resistance.	Target TNFSF14–TNFRSF14 axis to disrupt Treg‐CAF interaction and reverse anti‐PD‐1 resistance.	[187]
Inflammatory pathways	NLRP3 inflammasome pathway	Pan‐cancer (chemotherapy)	Inflammation activates NLRP3 inflammasome, driving inflammatory factor release, tumor development, and cisplatin resistance.	GSTO1‐1 inhibitors (C5‐1, 10u) inhibit NLRP3 activation and enhance chemotherapy sensitivity.	[194]
	cGAS‐STING pathway	Pan‐cancer (chemotherapy)	Inflammation‐mediated abnormal cGAS‐STING activation exacerbates irinotecan‐induced delayed diarrhea and reduces treatment tolerance.	Brusatol inhibits cGAS‐STING activity, regulates gut microbiota, alleviates side effects, and improves tolerance.	[195]
	TLR pathway	PDAC (chemotherapy), liver cancer	Inflammatory microenvironment modulates TLR4 (dual pro/antitumor effects, inconsistent clinical responses); chemotherapy stress induces TLR3 nuclear translocation PDAC, chemotherapy resistance).	Develop TLR profile‐specific agonists (combined with immunotherapy); interfere with TLR3 nuclear translocation to reverse resistance.	[188, 196]
	NF‐κB pathway	Hepatocellular carcinoma (immunotherapy)	Inflammation activates NF‐κB pathway, mediating inflammatory factor secretion and PD‐L1 expression (hepatocellular carcinoma immune escape).	Indole‐3‐carbinol inhibits NF‐κB p105 ubiquitination, downregulates PD‐L1, and combines with PD‐1 inhibitors to block escape.	[197]
	p21CIP1–CDK1/2 axis	Glioblastoma (chemotherapy)	Temozolomide‐induced glioblastoma senescence (accompanied by local inflammation); p21CIP1–CDK1/2 axis mediates G2 arrest/endoreplication (recurrence/resistance risks).	Target p21CIP1–CDK1/2 axis to reverse senescence‐associated resistance and reduce recurrence rate.	[198]
	JAK1‐c‐Myc pathway	PDAC (chemotherapy)	Chemotherapy stress induces inflammatory activation, mediating JAK1‐TLR3‐PRMT5‐c‐Myc pathway activation (chemotherapy resistance).	Inhibit JAK1‐c‐Myc pathway to reverse chemotherapy resistance.	[188]

In various tumor treatments, inflammation serves as a core link inducing resistance, weakening therapeutic effects by remodeling the immune microenvironment and activating abnormal signaling pathways. In immunotherapy, a Treg‐CAF immunosuppressive niche forms at the tumor margin in nonalcoholic fatty liver disease‐related hepatocellular carcinoma; TNFSF14‐TNFRSF14 signaling mediates their interaction, leading to anti‐PD‐1 treatment resistance [187]; TH17 cells inhibit epithelial chemokine expression via C/EBPβ, impeding immune cell infiltration; meanwhile, coexpression of NOS2/COX2 in ER‐negative breast cancer constructs an “immune desert,” both reducing immunotherapy sensitivity [188]. In chemotherapy, in cancers such as PDAC, chemotherapy stress induces JAK1‐mediated TLR3 phosphorylation and nuclear translocation; nuclear TLR3 activates the c‐Myc pathway via PRMT5, enhancing cancer cell invasiveness and inhibiting apoptosis, leading to chemotherapy resistance [188]; Temozolomide‐induced senescence of glioblastoma cells is accompanied by local inflammation; the p21CIP1–CDK1/2 axis mediates G2 arrest and endoreplication, becoming a hidden danger for recurrence and resistance [198]. In targeted therapy, PTEN deletion and CXCR2 overexpression drive enzalutamide resistance in castration‐resistant prostate cancer bone metastases; their abnormalities remodel the microenvironment by recruiting inflammatory suppressor cells such as MDSCs and Tregs, synergistically weakening targeted efficacy [189].

Abnormal recruitment and functional polarization of inflammatory cells are core causes of the tumor immunosuppressive microenvironment; targeted regulation of inflammatory cells can open new pathways for clinical treatment. In breast cancer, tumors reprogram bone marrow granulopoiesis, promoting the transformation of neutrophils to an immunosuppressive phenotype and accelerating metastatic spread. Anti‐IL‐1β therapy can reverse this reprogramming process, restore normal neutrophil function, and reduce lung metastasis risk [190]. For “cold tumors” (poorly immunogenic tumors), combination therapy with mitochondrial complex I inhibitors and TLR agonists can “educate” neutrophils to acquire antitumor properties: TLR agonists enhance neutrophil granule secretion and NADPH oxidase expression via NF‐κB signaling, while complex I inhibitors amplify oxidative damage. Together, they synergistically induce neutrophil‐mediated antitumor effects [199]. In lung tumors, acute inflammation can drive the transformation of macrophages to a tumor‐killing phenotype; by targeting and regulating the inflammatory characteristics of macrophages, their functions can be reprogrammed to enhance antitumor immunity, providing an auxiliary strategy for lung cancer immunotherapy [191].

Sustained activation of core inflammatory pathways is a key link in the cross‐association between inflammation and tumors; inhibiting pathway activity can effectively weaken the survival advantage of tumors. Activation of the NLRP3 inflammasome pathway drives the release of inflammatory factors and promotes tumor development. GSTO1‐1 inhibitors C5‐1 and 10u inhibit its activation by covalently binding to the enzyme's active site, reducing inflammatory responses in mice while enhancing cisplatin cytotoxicity to achieve chemotherapy sensitization [194]. Abnormal activation of the cGAS‐STING pathway is closely associated with chemotherapy side effects; brusatol can effectively alleviate irinotecan‐induced delayed diarrhea and improve treatment tolerance by inhibiting the activity of this pathway and regulating the intestinal microbiota [195]. The TLR pathway exerts dual effects in liver cancer: TLR4 can promote or inhibit tumor progression depending on the microenvironment. Developing agonists based on patients’ specific TLR profiles for combination with immunotherapy is expected to resolve inconsistent clinical responses and improve the precision of liver cancer treatment [196].

Imbalanced secretion of inflammatory factors is both a hallmark of inflammatory responses and a driver of tumor progression; targeted regulation of inflammatory factors can achieve the therapeutic goal of “reducing toxicity and enhancing efficacy.” As a classic immune activator, IL‐2 is limited in clinical application by toxicities such as vascular leak syndrome. The IL‐2/IL‐10 fusion molecule DK210 retains the ability to activate cytotoxic T cells and NK cells while restricting regulatory T cell expansion, completely decoupling immune activation from toxic reactions [192]. As a key chemokine, CXCL10 mediates TNFα‐induced tumor vascular leakage; CXCL10‐neutralizing antibodies can regulate this process to optimize intratumoral delivery of nanoparticle‐encapsulated chemotherapeutic drugs. Conversely, exogenous supplementation of CXCL10 can compensate for drug delivery defects in tumors with low pericyte coverage [193]. NF‐κB pathway‐mediated secretion of inflammatory factors is associated with immune escape; indole‐3‐carbinol downregulates PD‐L1 expression by inhibiting NF‐κB p105 ubiquitination, blocking immune escape in hepatocellular carcinoma and providing mechanistic support for combination with PD‐1 inhibitors [197].

Chronic inflammation can activate neurotransmitter pathways, neuropeptide signals, and neuroimmune circuits, induce abnormalities in the neuroregulatory system, and lay a pathological foundation for tumorigenesis and development. Under chronic inflammatory conditions, sustained inflammatory stimulation regulates neuro‐related signaling pathways through multiple dimensions, triggering functional disorders and abnormal remodeling of the neuroregulatory system. This process thereby provides a key pathological basis for tumorigenesis and malignant progression. Specifically, chronic inflammation significantly activates neurotransmitter secretion and conduction pathways, simultaneously upregulating the expression and activity of neuropeptide signaling molecules and inducing abnormal activation and imbalance of neuroimmune circuits. The synergistic disruption of these pathways and circuits impairs the homeostatic balance of the neuroregulatory system, ultimately promoting tumorigenesis and development by regulating processes such as cell proliferation, inflammatory microenvironment remodeling, and immunosuppression formation.

### 4.4 Novel Material Delivery

Traditional tumor diagnosis and treatment are often limited by poor targeting, weak immune response, and inflammatory interference [200, 201]. However, the emergence of novel biomaterials, nanomedicines, and engineered formulations is opening new paths for tumor diagnosis and treatment by precisely regulating the microenvironment, efficiently delivering drugs, and activating antitumor immunity. Biomimetic materials provide innovative support for tumor diagnosis and treatment by simulating or regulating key elements of the biological microenvironment. KBiF4@HSA nanoclusters based on human serum albumin integrate diagnostic and therapeutic functions: they not only enhance dual‐energy CT imaging contrast to improve breast cancer diagnosis accuracy but also scavenge elevated glutathione at tumor sites and promote reactive oxygen species generation. When combined with radiotherapy, they increase local X‐ray dose deposition, strengthen DNA damage effects, and cause no significant inflammatory responses [202]. 3D biomimetic collagen hydrogels have revealed the regulatory role of the ECM in immune function: aligned ECM matrices downregulate oxidative phosphorylation and reduce ATP production in immature DCs, impairing their migration ability and inhibiting immune activation by directly regulating T cell behavior. This suggests that targeted ECM structural remodeling may become an auxiliary strategy for tumor immunotherapy [203].

Nanomedicines excel in regulating the tumor inflammatory microenvironment and enhancing therapeutic effects due to their precise targeting and multifunctional integration. The self‐assembled nanomedicine FCP—composed of carminic acid, iron ions, and so forth—combines photoacoustic imaging‐guided photothermal therapy (PTT) with anti‐inflammatory activity. It can effectively alleviate PTT‐induced reactive oxygen species elevation and inflammatory responses, thereby inhibiting primary breast cancer growth and metastatic spread [204]. Sub‐1 nm CuO‐PMA nanosheets achieve controllable pyroptosis activation via ultrasound regulation: ultrasound triggers electron‐hole separation and the conversion of Cu(II) to Cu(I), efficiently generating reactive oxygen species and consuming glutathione. They induce pyroptosis via the caspase‐1/GSDMD axis, converting immunosuppressive “cold tumors” to “hot tumors” and significantly enhancing antitumor immune responses [205].

Intelligent delivery systems solve the inefficiency and toxicity issues of traditional therapies by overcoming physiological barriers and achieving targeted drug release. Dipeptide‐functionalized polymer nanocarriers can cross the blood–brain barrier, precisely targeting hypothalamic microglia. Delivering IRAK4 inhibitors effectively alleviates cancer cachexia‐related neuroinflammation, increasing food intake and reducing muscle loss by 50% in mice [206]. The “nanomedical firefighter” PsiL@M1M achieves spatiotemporal precise regulation of tumor treatment: it targets tumors and premetastatic niches via M1 macrophage membranes; after PTT destroys primary lesions, it blocks inflammation–metabolism crosstalk by neutralizing inflammatory factors and delivering siLDHA to interfere with lactate metabolism, reversing the immunosuppressive microenvironment and inhibiting tumor metastasis and recurrence [207].

**FIGURE 3:**
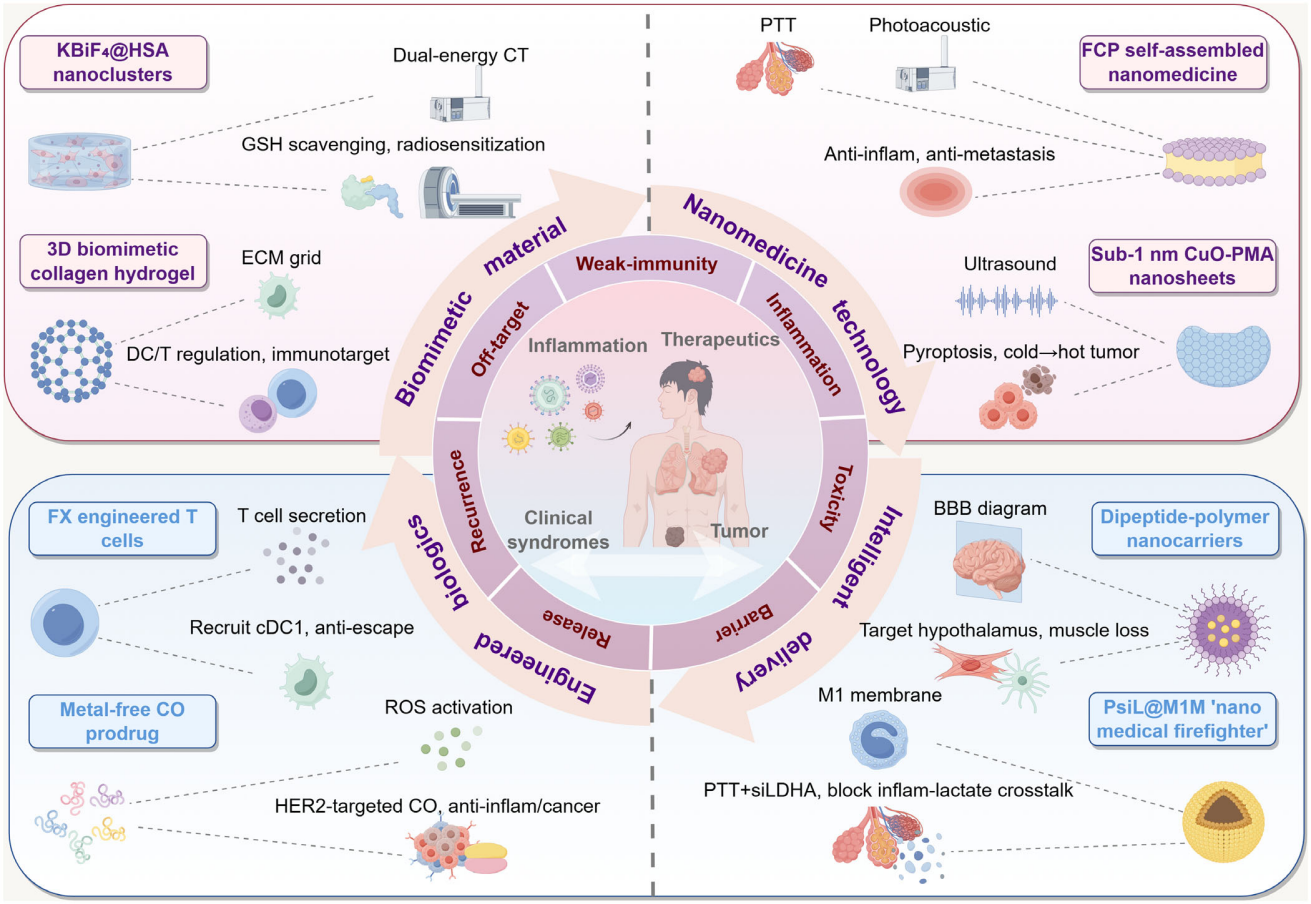
Novel material‐based delivery. Technical features: Nanomedicines, biomimetic materials, intelligent delivery systems, and engineered biological agents achieve synergistic anti‐inflammatory and antitumor therapy through precise targeting and functional integration.

Engineered biological agents construct efficient antitumor immune responses by modifying natural biomolecules or cells. FX‐engineered T cells are designed to secrete Flt3L and XCL1, which recruit and activate conventional type 1 dendritic cells (cDC1) while maintaining a stem cell‐like T cell pool. They trigger antigen spreading and endogenous polyclonal T cell responses, effectively recognizing heterogeneous antigen tumors and preventing immune escape [208]. Metal‐free CO prodrugs achieve targeted drug release by activation via reactive oxygen species enriched in the tumor microenvironment: when conjugated with trastuzumab, they can specifically release CO in HER2‐highly expressed tumor cells, exerting anti‐inflammatory and anticancer effects while avoiding metal carrier‐related toxicity, providing a new tool for tumor‐targeted therapy [209]. These innovative technologies have brought breakthroughs from “universal treatment” to “precision treatment” in tumor diagnosis and treatment, but challenges such as safety evaluation and large‐scale production still need to be addressed. With the development of multidisciplinary integration, they are expected to become core forces in precision medicine, injecting new hope into improving patient prognosis, as shown in Figure 3.

In summary, the exploration of therapeutic targets and development of drugs for inflammation‐related tumors have established a comprehensive technical chain, encompassing core links such as basic mechanism research, development of diagnostic auxiliary technologies, individualized optimization of treatment regimens, and advancement of drug delivery systems. Preclinical validation lays the foundation for target validation, auxiliary diagnostic factors enable precise stratification, combined treatment regimens break the limitations of single therapies, and novel material delivery systems improve efficacy and safety. This multidimensional research model not only deepens the understanding of the interaction between inflammation and tumors but also accelerates the clinical translation of anti‐inflammatory and antitumor drugs, providing strong support for the precision treatment of inflammation‐related tumors.

## 5 Conclusions and Prospect

The association between inflammation and tumors involves development is governed by a complex regulatory network consisting of multiple cell types and signaling pathways. Although recent studies have revealed the “internal driver–external attraction” regulatory framework that underlying inflammation‐mediated tumor initiation and progression, as well as the core pathways contributing to therapeutic resistance, the bidirectional regulatory mechanisms between these two components remain insufficiently elucidated, with inadequately understood. This core logic reflects manifested in two key principal dimensions: synchronous coordination and mutual causality. Specifically, internal drivers and external attractions do not follow a distinct temporal sequence activated in a sequential manner but are instead synchronously activated and dynamically coupled on the initiation interconnected during the onset of chronic inflammation to, form an uninterrupted regulatory loop, and they do not rely on a one‐way relationship but instead a continuous regulatory loop. Furthermore, these components do not operate through a unidirectional relationship; rather, they mutually trigger and amplify protumor effects, leading to a vicious self‐perpetuating cycle. This bidirectional regulatory pattern directly results in the limited efficacy significantly limits the effectiveness of single‐target interventions. Focusing solely on internal drivers allows the microenvironment to configure compensatory pathways through paracrine signaling. Conversely, targeting only external attractions enables tumor cells to rapidly switch allows tumor cells to swiftly alter their metabolic phenotypes via epigenetic memory, ultimately inducing to therapeutic resistance. Therefore, deciphering the core elucidating the fundamental molecular nodes and coupling rules of mechanisms governing this bidirectional regulation is crucial for clarifying the pathological underpinnings of inflammation‐induced tumorigenesis and provide. This understanding offers a novel theoretical basis for the development of synergistic “internal–external” synergistic targeted strategies to break the protumor cycle.

In terms of mechanism elaboration, future research should focus on the cell‐specific regulatory mechanisms of inflammatory signals: most existing studies rely on a “tumor cell–immune cell” binary model [210, 211], yet the crosstalk between inflammatory signals and stromal cells (e.g., fibroblasts, endothelial cells) or nerve cells in the tumor microenvironment remains unclear. For instance, how neurotoxicity affects tumor progression by regulating immune cell function via neurotransmitters, and how feedback regulation occurs between fibroblast cytokines and inflammatory signals during stromal fibrosis. These precise intercellular communication mechanisms are key to deciphering the “external attractions” of inflammation‐induced tumorigenesis and prerequisites for developing cell‐specific intervention strategies. Meanwhile, it is also necessary to leverage technologies such as single‐cell sequencing and spatial transcriptomics to explore the genetic and epigenetic heterogeneity of tumor cells across different inflammatory stages (acute/chronic), clarify the role of “inflammatory memory” in tumor recurrence, and identify new targets to block the “memory transmission” of inflammation‐induced tumorigenesis.

In the field of therapeutic development, personalized anti‐inflammatory–antitumor combination strategies will become the core direction. Current combined therapies mostly rely on universal regimens of “anti‐inflammatory agents + immune checkpoint inhibitors,” but inflammation‐driven pathways vary significantly among different inflammation‐related tumors (e.g., CAC, hepatitis‐associated liver cancer). It is therefore necessary to screen tumor‐specific inflammatory markers (e.g., specific cytokines, microbial metabolites) via multiomics analysis to achieve precise combined therapy with “patient stratification–target matching.” In addition, the development of novel material delivery systems needs to overcome the triple bottlenecks of “targeting–safety–long‐acting efficacy”: on one hand, technologies such as engineered small extracellular vesicles and smart responsive nanocarriers can be used to achieve synergistic targeted delivery of anti‐inflammatory agents and antitumor drugs, reducing inflammatory damage to normal tissues; on the other hand, it is necessary to optimize the pharmacokinetics and biocompatibility of delivery systems in line with clinical translation needs, promoting more basic research findings into Phase I/II clinical trials.

The improvement of auxiliary diagnosis and prognostic evaluation systems is also critical. Existing auxiliary diagnostic factors mostly focus on single indicators (e.g., NLR [212], GDF15 [213]). In the future, it is essential to construct multidimensional “inflammation–tumor” combined diagnostic models that integrate multiomics data (e.g., immunoinflammatory indicators, microbial community characteristics, epigenetic markers such as circRNA and DNA methylation). Machine learning algorithms can be used to improve diagnostic sensitivity and specificity, enabling early warning and prognostic stratification of inflammation‐related tumors. At the same time, large‐scale prospective clinical studies should be conducted to validate the clinical value of novel diagnostic factors and promote their transition from laboratory research to routine clinical application. Moreover, the dual nature of inflammation in tumor regulation cannot be ignored: its “antitumor potential” can be realized through controlled activation—for example, mast cells enhance the antitumor activity of MAIT cells via inflammasome activation [214], exercise‐induced vesicles remodel the immune microenvironment of triple‐negative breast cancer [215], FLASH radiotherapy reprograms the inflammatory properties of macrophages [216], and strategies such as STING agonist conjugates can also precisely activate antitumor inflammation [217]; whereas its “protumor risk” stems from dysregulation—for example, cancer cells disrupt ZNF93 to disturb the balance between inflammation and genomic stability [218], abnormal WSTF nuclear autophagy drives chronic inflammation‐related tumors [219], and excessive inflammation may promote tumor metastasis or induce immunosuppression [220]. In the future, balancing these two aspects requires focusing on “precision regulation”: elaborating the molecular characteristics of these two types of inflammation to develop targeted tools, and combining modular delivery with tumor‐tailored regimens to maximize the antitumor effect of inflammation while inhibiting protumor risks.

In addition, research on inflammation and tumors needs to strengthen interdisciplinary collaboration, integrating technologies and theories from fields such as immunology, microbiology, neuroscience, and materials science to decipher the overall regulatory network of inflammation‐induced tumorigenesis from a “systems biology” perspective. For example, microbiomics can be used to screen probiotic communities that regulate inflammation, and combined with synthetic biology techniques to modify engineered bacteria for targeted anti‐inflammation; or neuromodulation technologies (e.g., vagus nerve stimulation) can be used to intervene in the neuro–immune–tumor axis, providing new paradigms for the treatment of inflammation‐related tumors. In summary, by in‐depth elaboration of precise regulatory mechanisms, development of personalized therapeutic strategies, and improvement of diagnostic evaluation systems in the future, it is expected to break through current treatment bottlenecks, drive the efficient transition of this field from “mechanism research” to “clinical translation,” and ultimately improve patient prognosis and quality of life.

## Author Contributions

Xiaodie Liu and Ziyuan Wang contributed equally to this work and should be considered as cofirst authors. Yicun Han and Qing Ji jointly supervised this work. Xiaodie Liu and Ziyuan Wang wrote the main manuscript text. Huirong Zhu was responsible for completing all the figure‐making work in the paper. All the authors reviewed and approved the final manuscript.

## Funding

This work was supported by grants from National Natural Science Foundation of China (82274297, 82074225, 82030118, 82374188).

## Ethics Statement

The authors have nothing to report.

## Conflicts of Interest

The authors declare no conflicts of interest.

## Data Availability

The authors have nothing to report.
